# MoG+: a database of genomic variations across three mouse subspecies for biomedical research

**DOI:** 10.1007/s00335-021-09933-w

**Published:** 2021-11-15

**Authors:** Toyoyuki Takada, Kentaro Fukuta, Daiki Usuda, Tatsuya Kushida, Shinji Kondo, Shoko Kawamoto, Atsushi Yoshiki, Yuichi Obata, Asao Fujiyama, Atsushi Toyoda, Hideki Noguchi, Toshihiko Shiroishi, Hiroshi Masuya

**Affiliations:** 1 Integrated Bioresource Information Division, RIKEN BioResource Research Center, 3-1-1 Koyadai, Tsukuba, Ibaraki 305-0074 Japan; 2 Center for Genome Informatics, Joint Support-Center for Data Science Research, Research Organization of Information and Systems, 1111 Yata, Mishima, 411-8540 Japan; 3 Advanced Genomics Center, National Institute of Genetics, 1111 Yata, Mishima, 411-8540 Japan; 4 Genetic Informatics Laboratory, National Institute of Genetics, 1111 Yata, Mishima, 411-8540 Japan; 5 Experimental Animal Division, RIKEN BioResource Research Center, 3-1-1 Koyadai, Tsukuba, Ibaraki 305-0074 Japan; 6 RIKEN BioResource Research Center, 3-1-1 Koyadai, Tsukuba, Ibaraki 305-0074 Japan; 7 Comparative Genomics Laboratory, National Institute of Genetics, 1111 Yata, Mishima, 411-8540 Japan

## Abstract

Laboratory mouse strains have mosaic genomes derived from at least three major subspecies that are distributed in Eurasia. Here, we describe genomic variations in ten inbred strains: *Mus musculus musculus*-derived BLG2/Ms, NJL/Ms, CHD/Ms, SWN/Ms, and KJR/Ms; *M*. *m*. *domesticus*-derived PGN2/Ms and BFM/Ms; *M*. *m*. *castaneus*-derived HMI/Ms; and JF1/Ms and MSM/Ms, which were derived from a hybrid between *M*. *m*. *musculus* and *M*. *m*. *castaneus*. These strains were established by Prof. Moriwaki in the 1980s and are collectively named the “Mishima Battery”. These strains show large phenotypic variations in body size and in many physiological traits. We resequenced the genomes of the Mishima Battery strains and performed a comparative genomic analysis with dbSNP data. More than 81 million nucleotide coordinates were identified as variant sites due to the large genetic distances among the mouse subspecies; 8,062,070 new SNP sites were detected in this study, and these may underlie the large phenotypic diversity observed in the Mishima Battery. The new information was collected in a reconstructed genome database, termed MoG+ that includes new application software and viewers. MoG+ intuitively visualizes nucleotide variants in genes and intergenic regions, and amino acid substitutions across the three mouse subspecies. We report statistical data from the resequencing and comparative genomic analyses and newly collected phenotype data of the Mishima Battery, and provide a brief description of the functions of MoG+, which provides a searchable and unique data resource of the numerous genomic variations across the three mouse subspecies. The data in MoG+ will be invaluable for research into phenotype-genotype links in diverse mouse strains.

**Supplementary Information:**

The online version contains supplementary material available at 10.1007/s00335-021-09933-w.

## Introduction

The genomes of the classical laboratory mouse strains have mosaic structures with discrete segments originating from at least three genetically divergent subspecies, namely, *Mus musculus domesticus*, *M*. *m*. *musculus*, and *M*. *m*. *castaneus* (Frazer et al. 2007; Keane et al. 2011); these subspecies originate from geographically isolated regions of Eurasia (Moriwaki 1994; Silver 1995). It is now well established that the genomes of the classical inbred strains are overwhelmingly derived from the Western European subspecies *M*. *m*. *domesticus*, with the remaining sequences mostly derived from the Japanese subspecies *M*. *m*. *molossinus* (Yang et al. 2011). *M*. *m*. *molossinus* is a hybrid of two subspecies, *M*. *m*. *musculus* and *M*. *m*. *castaneus* (Yonekawa et al. 1980), although its genome is predominantly derived from the former (Yonekawa et al. 1988; Bonhomme et al. 1989). Kazuo Moriwaki and colleagues at the National Institute of Genetics (NIG) in Japan established a series of wild-derived inbred mouse strains collectively named the “Mishima Battery” (Fig. S1) (Shiroishi 2014). These strains were derived from subspecies that have been isolated for a considerable time in the wild and exhibit a wide range of differentiated phenotypes as a consequence of adaptation to different environments (Moriwaki 1994; Moriwaki et al. 2009). The strains of the Mishima Battery are maintained as breeding stocks at the NIG with complete pedigree records of successive inbreeding generations. The behavioral phenotypes of some strains have been reported (Koide et al. 2000; Furuse et al. 2002).

Previously, we performed whole-genome resequencing of two *M*. *m*. *molossinus*-derived strains, MSM/Ms (hereafter referred to as MSM) and JF1/Ms (JF1) that are members of the Mishima Battery. A comparative genomic analysis of the strains and a reference strain C57BL/6 J (B6) suggested that the ancestor of the JF1 strain provided the *M*. *m*. *molossinus* genome in classical laboratory strains and largely contributed to its intersubspecific genome diversity (Takada et al. 2013). Information on the comparative genomic variations was made available in a public database named NIG_MoG (Takada et al. 2015). The data on single nucleotide polymorphisms (SNPs) in MSM and JF1 has been widely used to identify allele-specific markers for epigenetic and gene expression analyses in commonly used laboratory strains (Yagi et al. 2017, 2019; Kondo et al. 2019).

We have now extended genome resequencing to all strains of the Mishima Battery (*M*. *m*. *musculus*-derived BLG2/Ms (BLG2), NJL/Ms (NJL), CHD/Ms (CHD), SWN/Ms (SWN) and KJR/Ms (KJR); *M*. *m*. *domesticus*-derived PGN2/Ms (PGN2) and BFM/Ms (BFM); *M*. *m*. *castaneus*-derived HMI/Ms (HMI); and JF1 and MSM) and conducted a comprehensive comparative genomic analysis across these mouse strains. A simple presentation of the short-reads from the sequenced Mishima Battery strains with mapping to the reference sequence was first made available in 2018, and the data was released as an updated database (NIG_MoG2).

In 2019, operation of this database was transferred to the RIKEN BioResource Research Center (BRC) (Masuya et al. 2021), and the database was renamed “MoG+” (https://molossinus.brc.riken.jp/mogplus/). In this study, we performed a new comparative genomics analysis with dbSNP data (Mouse dbSNP 142; ftp://ftp-mouse.sanger.ac.uk/). Our analyses identified 8,062,070 new SNP sites in the 81,866,820 nucleotide coordinates at variant sites following the addition of the Mishima Battery data to the dbSNP data. The MoG+ database allows visualization of the genome-wide pattern of intersubspecific nucleotide variants across both the Mishima Battery and some commonly used laboratory mouse strains. The web browser can be used to retrieve information on nucleotide variants from genomic regions of interest and in genomic segments from specific subspecies in the classical laboratory strains. This feature enables exploration of genes involved in disease susceptibility and phenotypic variation in genetic studies using intersubspecific crosses. MoG+ stores a wide range of data on genomic variations that cannot be captured by comparison among the standard laboratory strains. Since different human ethnic populations contain considerable genomic diversity, which has an influence on individual phenotypes, the mouse intersubspecific nucleotide variants will be a useful source for identifying novel genomic variations responsible for differences in phenotypes and disease susceptibilities. We have therefore added links from MoG+ to other public databases related to human diseases and phenotypes. In addition, we have added a catalog of experimental mouse strains that are available from RIKEN BRC.

In this report, we provide a systematic collection of morphological and physiological phenotypic data of the strains of the Mishima Battery. Furthermore, we present the results of the comparative genomic analysis from resequencing the genomes of the Mishima Battery strains, and also give a brief introduction to the new functions of MoG+, focusing on software and viewer application changes. The new data and the updated genome database will be of value for future research into linkages between phenotypes and nucleotide variants in the Mishima Battery and classical laboratory strains and for elucidation of gene functions relevant to phenotypic diversity.

### Subspecies-specific phenotypes of the Mishima Battery

**Fig. 1:**
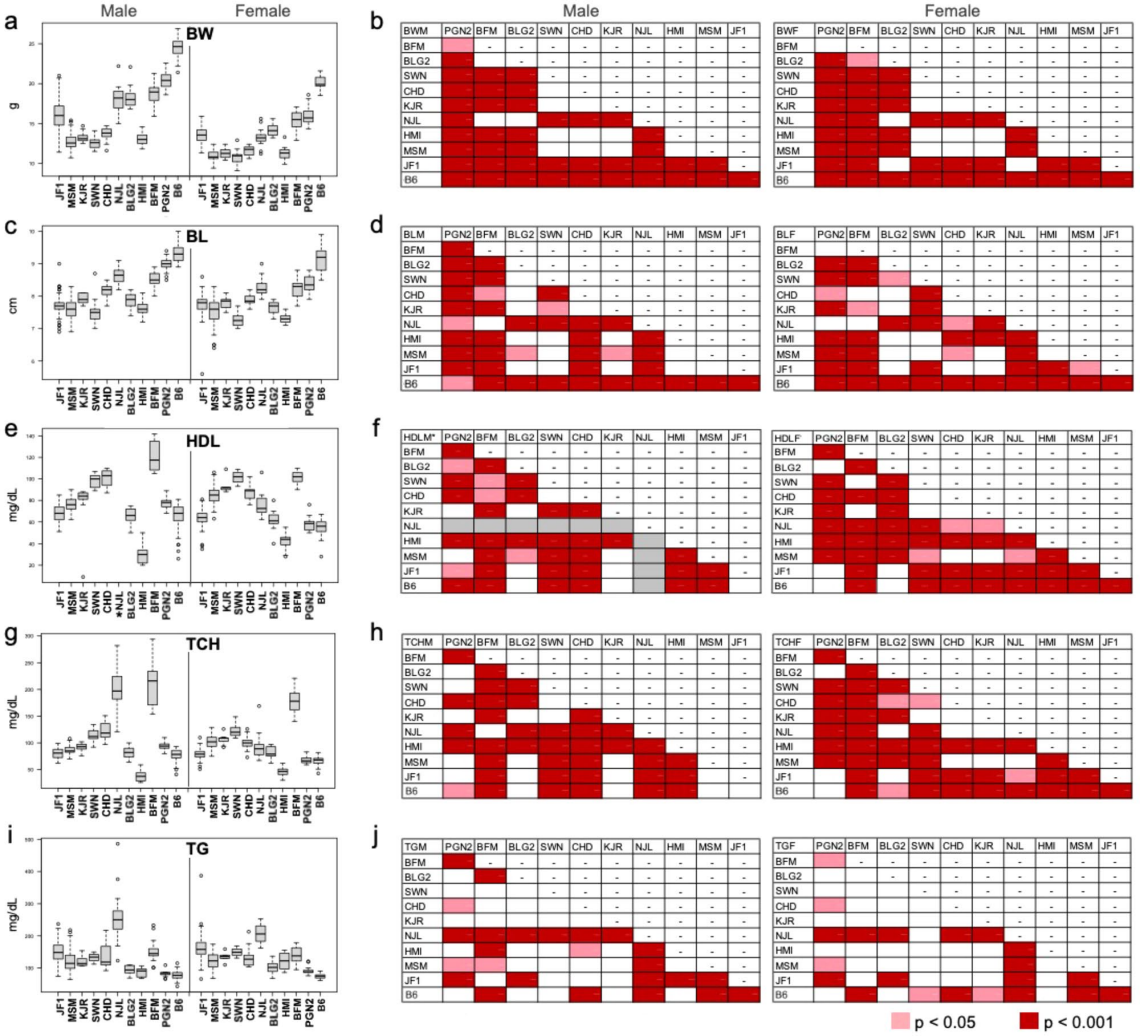
Representative phenotypes of the Mishima Battery. The phenotypes are depicted by Boxplots (**a**, **c**, **e**, **g**, **i**) and trait-strain matrices (**b**, **d**, **f**, **h**, **j**). In the trait-strain matrices, a measured value was considered significant if the corrected *t*-test *p* value was less than 0.05 (pale red) and highly significant if the *p* value was less than 0.001 (red). (**a**), (**b**) Body weight (BW); (**c**), (**d**) Body length (BL: nose-to-anus distance); (**e**), (**f**) Blood concentration of high-density lipoprotein (HDL), (**g**), (**h**) total cholesterol (TCH), and (**i**), (**j**) triglyceride (TG). *Blood levels of HDL for the NJL males are not shown because the values exceed the measurable threshold range (corresponding rows and columns are greyed)

To expand the value of MoG+ for biomedical research, we systematically phenotyped the Mishima Battery strains as well as B6 (Fig. S1; Table S1). The phenotypic measurements mainly focused on body size and physiological traits. The raw measurement data is downloadable from the RIKEN BRC website (https://molossinus.brc.riken.jp/pub/Wild10_2020/phenotype_mishimabattery_2021.xlsx). Mice (9–11 weeks of age; 67–79 days after birth) were used for the data collection, which is summarized in Fig. 1 and Figure S2. If the measured value of a trait in one strain significantly differed from those of the remaining strains, the strain was marked in a traits-strains matrix (Figs. 1 and S3).

The Mishima Battery strains show a wide range of phenotypes with regard to morphological and physiological traits. In cross comparisons of the Mishima Battery strains with B6, the rate of each pair that indicates statistically significant values was 61.9% with *p* < 0.001 and 6.4% with *p* < 0.05 for a total of 1145 comparisons for males, and 53.5% with *p* < 0.001 and 9.5% with *p* < 0.05 for a total of 1100 comparisons for females. Several notable phenotypes are summarized in Fig. 1: the Mishima Battery strains had a lower average body weight (BW) and shorter body length (BL) than the B6 strain (Fig. 1 a–d). The MSM, SWN, CHD, KJR, and HMI strains, which originated from East Asia, have a lower BW than the PGN, BFM, BLG2, and NJL strains, which originated in Europe (Fig. 1a). Seven clinical biochemical parameters were measured in blood samples, including lipid metabolism-related components, enzyme activities, and other protein and mineral components that are good indicators of physiological phenotypes. The Mishima Battery strains had a higher level of high-density lipoprotein (HDL), total cholesterol (TCH), and triglyceride (TG) than the B6 strain in both males and females (Figs. 1e–j and S2). It is notable that the level of HDL in the blood of NJL males was extremely high and exceeded the measurable threshold range (https://molossinus.brc.riken.jp/pub/Wild10_2020/phenotype_mishimabattery_2021.xlsx). As described above, genomic information on the Mishima Battery strains is extremely useful because they have a wide variety of phenotypes, some of which might be related to human disease-related phenotypes such as fat accumulation and blood chemistry values. In addition, the phenotype data from the Mishima Battery strains may also provide insights into genomic variations that might underlie phenotypic variations in mouse inbred strains with a *M*. *m*. *molossinus*-derived mosaic genome.

### Resequencing data from Mishima Battery strains

**Fig. 2:**
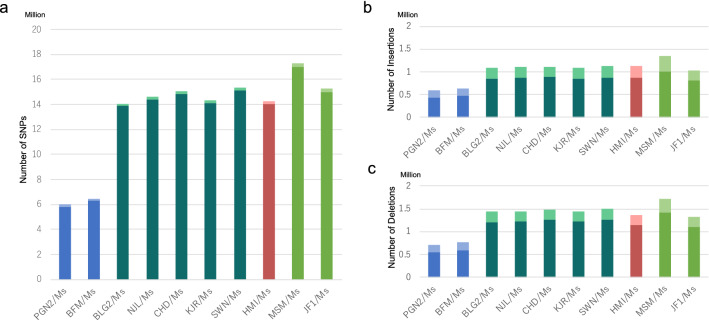
Number of nucleotide sequence variants in each Mishima Battery strain compared to B6. The numbers of SNPs (**a**), insertions (**b**), and deletions (**c**) are shown. The subspecies from which the Mishima Battery strains were derived are shown by different colors: *Mus m*. *domesticus*, blue; *Mus m*. *musculus*, green; *Mus m*. *castaneus*, auburn; and *Mus m*. *molossinus*, pea green. In the bar charts, the number of heterozygous SNPs is indicated by the lighter color and of homozygous SNPs by the darker color

The resequencing of the genome of the Mishima Battery MSM strain updated the data on nucleotide variants by the addition of 78.9-fold short-reads to the previously reported data set based on 2.4-fold capillary sequencing and 16.5-fold short-reads (Takada et al. 2013). This update therefore significantly improved the quality of the MSM genome data. The nucleotide variants information obtained from the resequencing and the comparative genomic analysis are summarized in Fig. 2 and Table S3. Comparison of the resequenced genome with the reference B6 genome (GRCm38/mm10) identified SNPs in the range 5,999,933–17,305,163, insertion variants in the range 596,123–1,340,886, and deletion variants in the range 714,633–1,723,300 (Table S3). This study increased the number of homozygous SNPs detected by 35% from 12,539,294 (Takada et al. 2013) to 16,974,847 (Table S3). This facilitates the use of the SNP data in epigenetic studies and allele-specific gene expression analyses since the upgraded nucleotide variants data between MSM and B6 yields at least one SNP in each coding sequence of most protein-coding genes for use as an allele-specific marker. The lowest rate of variants was found in the comparison with PGN2 and the maximum with MSM. In total, 45,093,250 SNP sites were identified. Table 1 shows the SNP frequency for the whole-genome between the reference B6 genome and all strains of the Mishima Battery, and also shows SNP frequencies in cross comparisons among the Mishima Battery. As expected, intrasubspecific comparisons yielded low values (0.15–0.33%), whereas intersubspecific comparisons yielded higher values (0.71–0.88%). A dendrogram was constructed from the whole SNP dataset (strain name.snp.tsv; https://molossinus.brc.riken.jp/pub/Wild10_2020/) to summarize the relationships between the strains in the Mishima Battery (Figure S4, Table S4). Low-quality regions around indels were excluded in this analysis.

**Table 1:** Percent of SNP variants between the reference B6 strain and Mishima battery strains, and cross comparison between each strain of the Mishima Battery^*^^†^

Strain	PGN2/Ms	BFM/Ms	BLG2/Ms	NJL/Ms	CHD/Ms	KJR/Ms	SWN/Ms	HMI/Ms	MSM/Ms	JF1/Ms
Subspecies	*domesticus*		*musculus*					*castaneus*	*molossinus*	
C57BL/6J (mm10)	0.27	0.3	0.7	0.7	0.73	0.7	0.74	0.71	0.82	0.75
Cross comparison	PGN2/Ms	BFM/Ms	BLG2/Ms	NJL/Ms	CHD/Ms	KJR/Ms	SWN/Ms	HMI/Ms	MSM/Ms	JF1/Ms
PGN2/Ms										
BFM/Ms	0.27									
BLG2/Ms	0.76	0.76								
NJL/Ms	0.76	0.76	0.33							
CHD/Ms	0.77	0.8	0.34	0.33						
KJR/Ms	0.78	0.78	0.34	0.32	0.22					
SWN/Ms	0.81	0.81	0.33	0.32	0.21	0.15				
HMI/Ms	0.75	0.76	0.74	0.71	0.72	0.72	0.73			
MSM/Ms	0.88	0.88	0.36	0.35	0.24	0.2	0.18	0.76		
JF1/Ms	0.82	0.82	0.36	0.37	0.26	0.22	0.21	0.72	0.17	

*Low-quality regions around indels were excluded in this analysis

†“Percent of SNP variants” is the ratio of total SNPs to effective total genome length between the two strains (Table S3)

### Annotation of SNPs and short indels reveals variation in gene features

**Fig. 3:**
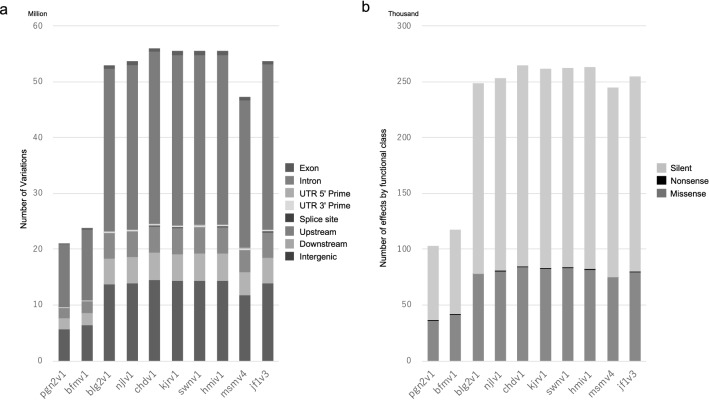
Genomic variants in Mishima Battery strains compared to B6. Numbers of genomic variants in specific genomic locations (**a**) and functional classes for the effects of amino acid substitutions (**b**) are shown

Nucleotide variant datasets include SNPs and short indels for each strain of the Mishima Battery, named pgn2v1 (PGN2), bfmv1 (BFM), blg2v1 (BLG2), njlv1 (NJL), chdv1 (CHD), kjrv1 (KJR), swnv1 (SWN), hmiv1 (HMI), msmv4 (MSM), and jf1v3 (JF1), respectively, were annotated using SnpEff (Cingolani et al. 2012) to provide genomic location and functionality based on the Genome Reference Consortium Mouse Build 38 (GRCm38.82) (http://ensembl.org/index.html). Low-quality regions around indels were included in this analysis in order to store the maximum amount of genome variant information in MoG+. The variant classification is depicted in Fig. 3a. Briefly, an average of 1.24% variants (range 1.22–1.31% for the Mishima Battery), 54.64% (53.10–55.91%) in introns, 25.91% (24.68–26.82%) in intergenic regions, 0.10% (0.101–0.105%) in splicing regions, 0.12% (0.112–0.129%) in 5′ untranslated regions (UTRs), and 0.59% (0.58–0.64%) in 3′ UTRs in overlapping variant sites were identified (Fig. 2a). Exonic variants were further classified on the basis of variant type in the all-variants set: an average of 0.22% (0.173–0.311%) NONSENSE (stop) variants was identified, 31.13% (30.35–35.38%) of MISSENSE (nonsynonymous) variants, and 67.64% (64.33–69.38%) of SILENT (synonymous) variants. The classification of the exonic variants is depicted in Fig. 3b. A summary of the annotation process for the variant classification is presented in Table S5. Our nucleotide variant dataset was also compared with the publicly available dataset (Mouse dbSNP 142; ftp://ftp-mouse.sanger.ac.uk/). The compilation of the MoG+ data and the Mouse dbSNP 142 showed that a total of 81,866,820 sites, corresponding to about 3% of the mouse genome (mm10), was polymorphic (Table S6). The inclusion of the MoG+ data in the comparison increased the number of SNPs by 9.8%. The subspecies-specific SNPs are also listed in Table S6. We observed large differences in the number of variants across strains even within the same subspecies. For example, the comparison of the *castaneus*-derived strains HMI and CAST/EiJ identified 14,427,289 SNPs, a frequency equivalent to that found in comparisons between subspecies (Table S3).

### Enhancement of software applications and new viewers

Web-based genome browsers are usually categorized into general genome browsers and species-specific genome browsers. The Ensembl Genome Browser (Howe et al. 2021), NCBI Genome Data Viewer (GDV) (Rangwala et al. 2021) and UCSC Genome Browser (Gonzalez et al. 2021) are commonly used as general genome browsers for multiple species. MoG+ is a unique genome browser that focuses on the mouse genome, and which provides a graphical interface to browse, search, retrieve, and analyze mouse genomic variations. Compared to the general genome browsers, MoG+ does not allow comparison of interspecies genomic diversity or browsing across species, but it does allow quick search for nucleotide variants and amino acid substitutions in genes among commonly used experimental mouse strains. Via MoG+ , users are able to compare nucleotide variants in common laboratory mouse strains using genotype variation among the strains of the Mishima Battery as tag-SNPs, which reflect pure subspecies. This allows us to confirm whether a SNP in a given strain is a variant specific to a subspecies or a variant occurring specifically in that strain. This is extremely important because the information facilitates identification of causative SNPs in the region responsible for the phenotype of interest. MoG+ also enables use of gene symbols as hubs to search for human disease information and mouse models available from RIKEN BRC.

### Introduction to MoG+ 

**Fig. 4:**
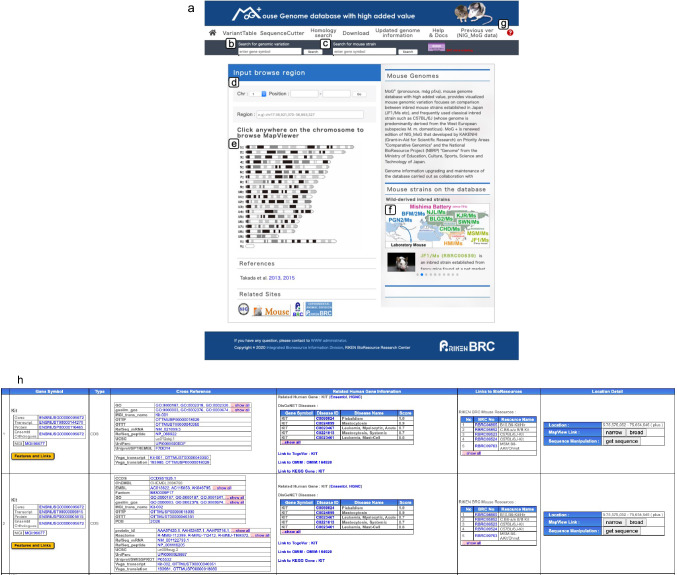

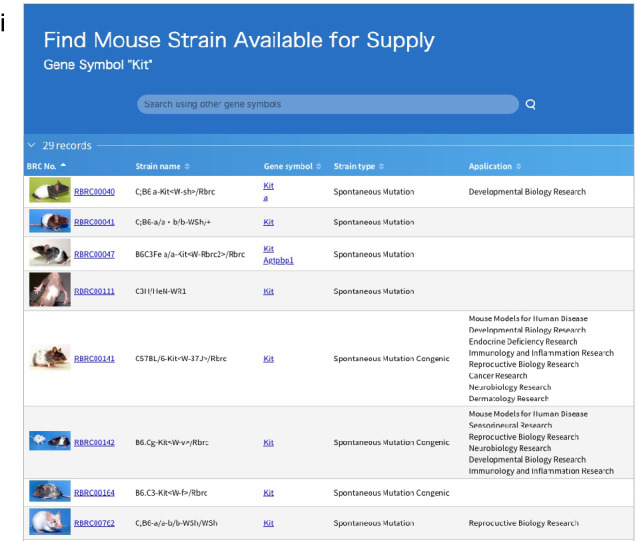
Screenshot of the top page of MoG+ showing the different functions available (**a**). The window named “Search for genomic variation” (**b**) is the entrance to information on genomic variations in Mishima Battery mouse strains and to information from gene searches; the information is retrieved from other public databases. For this search window, gene symbols are used as the search query. The search window “Search for mouse strain” enables one to find experimental mouse strains that are available from RIKEN BRC (**c**). In this search window, gene symbols are used as the search query. The “Chromosome and position-interval” search window is a gateway to “Map View”, a page displaying information on the distribution of genes in the designated chromosomal area (**d**). It is also possible to access this information by use of the chromosome ideogram (Mouse Genome Sequencing Consortium 2002) (**e**). The genomic region for a search query can be defined by placing the cursor in the required location of the chromosome ideogram. Geographical distribution of the ancestral subspecies of the Mishima Battery strains (**f**). Link to the tutorial for MoG+ (**g**). A gateway page named “Search result” opens following a query using the “Search for genomic variation” window; the illustrated example was produced using “Kit” as the search query (**h**). In this page, the first column on the left-hand side shows the search result numbers. The second column named “Gene Symbol” shows gene symbols in the database containing perfect and partial matches of the text search, and links to their IDs in the Ensembl and JAX databases. The yellow button labeled “Features and Links” links to a gateway page where the user can obtain genomic variation information on the searched gene in the database. The third column named “Type” shows the type of data available on the searched gene as gene, mRNA, and coding sequence (CDS). The fourth column named “Cross Reference” shows links to the searched gene in public databases. The fifth column named “Related Human Gene Information” shows human disease information related to the searched gene based on the match of the text search of the gene symbol. The sixth column, “Links to BioResources”, shows links to experimental mouse strains that are available from RIKEN BRC. The last column, “Location Detail”, shows the region on the chromosome where the searched gene is located. This column also contains links to “Map View” for “narrow” (< 100 kb) or “broad” (< 300 kb) ranges. “Sequence Manipulation” is linked to the “SequenceCutter” function. An example of the search results of using “Search for mouse strain” obtained using “Kit” as the search query (**i**)

The top page of MoG+ is shown in Fig. 4a. The update to the database has added new features; thus, all strains in the Mishima Battery are now included along with nucleotide variant information. We have also added new software applications to expand data manipulation in MoG+ that will enable data acquisition and editing of nucleotide sequences. MoG+ has links to various public databases: DisGeNET (Piñero et al. 2020), a search platform containing one of the largest publicly available collections of genes and nucleotide variants associated with human diseases; OMIM (https://omim.org/), an online catalog of human genes and genetic disorders; and ‘TogoVar’ (https://togovar.biosciencedbc.jp), a publicly accessible and comprehensive database of genetic variants in the Japanese population. These various links enable users to connect data on mouse genomic variations with those of human diseases and phenotypes. On the top page of MoG+ , a search by gene symbol retrieves information on human diseases, which are caused by a defect in the gene of interest (Fig. 4b). MoG+ has been updated to include a function to identify mouse strains from RIKEN BRC that carry mutations of a gene of interest (Fig. 4c). The chromosome and position-interval search window (Fig. 4d) and the chromosome illustration (Fig. 4e) are gateways for accessing chromosomal and genomic regions. The geographical range of the subspecies from which each Mishima Battery strain originated is shown on the world map (Fig. 4f). In addition, instructions for functions carried over from NIG_MoG are updated and provided in a tutorial (Fig. 4g; https://github.com/ttakada1/MoGplus_tutorial_2020/wiki). The function to search for gene annotations which includes links to the human diseases information has also been improved (Fig. 4h). Another new function termed “Search for mouse strain” can be used to retrieve strains as disease models available from RIKEN BRC (Fig. 4i). The top page of MoG+ also provides access to a function called “SequenceCutter” that has been retained from NIG-MoG (Takada et al. 2015). Users can retrieve and edit arbitrary pseudo-sequences of all Mishima Battery strains using SAMtools pileup functions (Li et al. 2009).

**Fig. 5:**
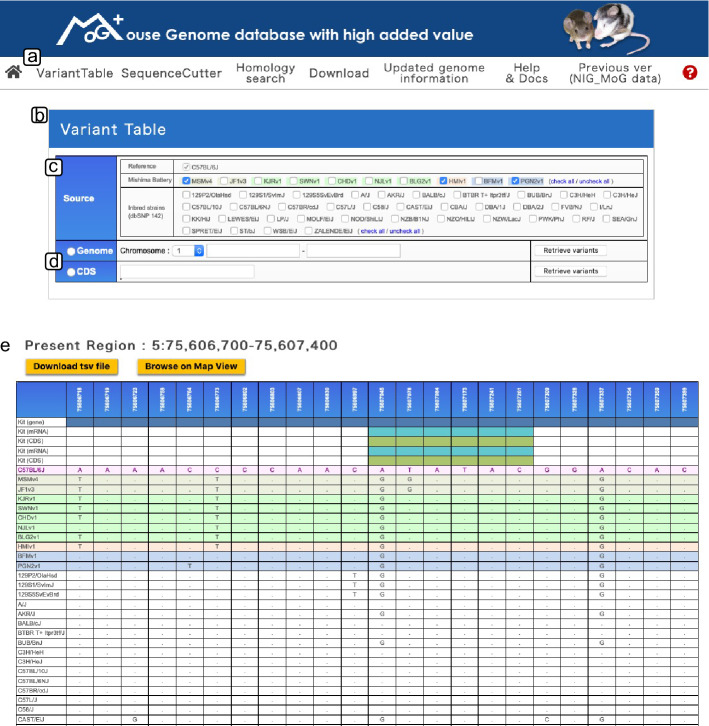
The “VariantTable” function is accessed from the common header present in MoG+ (**a**). When the “VariantTable” browser link is clicked, a gateway page is opened where the user can select which strains and genomic regions to display (**b**). “Source” can be used to obtain SNP information on searchable mouse strains: the reference B6 strain; Mishima Battery strains; and 36 selected strains from public databases (dbSNP) (**c**). The “VariantTable” function supports searches of genome coordinates and gene symbol-based SNP searches and provides access to any region of the genome of interest (**d**). An example of search results using the “Kit” gene region (chromosome 5: 75,606,700–75,607,400) as the search query (**e**). In this page, the first column on the left-hand side indicates the type of data available (gene, mRNA and CDS) and the user-selected strains. The genomic coordinates on each chromosome are indicated at the top of each column, with the most proximal in the first column on the left side. The information in the columns is highlighted according to the data type: gene, blue; mRNA, Turkish blue; CDS, pea green. The seventh line labeled “C57BL/6J” and highlighted in magenta shows reference SNPs. The rows for Mishima Battery strains are colored blue, green, vermilion, and moss green, for *M*. *m*. *domesticus*, *M*. *m*. *musculus*, *M*. *m*. *castaneus*, and *M*. *m*. *molossinus*, respectively. In this page, where nucleotides of the searched strain differ from the reference, the substituted nucleotide is displayed. A period (full stop) is displayed where the nucleotide is the same as the reference; the label “n.d.” is displayed when no data is available

To further expand the functionality of MoG+, we added a SNP search function entitled “VariantTable” (Fig. 5a), to enable users to retrieve SNPs for any specified genomic region in all Mishima Battery strains, B6, and 36 selected inbred mouse strains registered in the dbSNP database (Mouse dbSNP 142; ftp://ftp-mouse.sanger.ac.uk/). Data retrieval using VariantTable (Fig. 5b) is carried out by use of the appropriate search terms in the “Chromosome and position-interval” search window (Fig. 5c, d). The results page of VariantTable shows the gene name and exonic regions with various ranges of nucleotide sequences from single nucleotide to maximum 300 kbp and provides information on SNPs (Fig. 5e). VariantTable makes it simple to determine whether a SNP in a given mouse strain is unique to that strain or is shared with other strains belonging to the same subspecies. Comparison of strains bearing a phenotype of interest with the distribution pattern of a nucleotide variant will narrow down candidate genomic regions that might underlie the phenotype (Fig. 5e). VariantTable searches can also be used to design DNA sequences for producing specific genetic modifications using genome editing methods. Additionally, it is possible to download the search results as a CSV file, and a function on the browser to jump to genomic regions by selecting the coordinates of designated SNPs.

### Usability of MoG+ in genetic studies involving intersubspecific crosses

In 2002, a whole-genome draft sequence of the inbred strain B6 was reported by the Mouse Genome Sequencing Consortium (2002). Subsequently, numerous other mouse strains have been sequenced and can be used as reference strains (Lilue et al. 2018). The B6 reference sequence has been constantly updated (https://www.ncbi.nlm.nih.gov/grc/mouse/data; Sarsani et al. 2019).

Genetic factors that influence human phenotype and disease susceptibility have been successfully identified by studies using laboratory mouse strains (Munoz-Fuentes et al. 2018; Nadeau & Auwerx 2019; Cacheiro et al. 2020). Collections of inbred mouse strains have been established from the standpoint of intersubspecific genomic differences, such as chromosome substitution (CS or consomic) strains, collaborative cross (CC) strains, and diversity outbred (DO) strains. Each of these provides a powerful platform for mapping and assigning genomic regions that affect complex genetic traits (Nadeau et al. 2012; Saul et al. 2019). For genetic studies using these mouse strains, information on intersubspecific nucleotide variants and subspecies-derived genomic segments (blocks) is vital to deciphering relationships between haplotypes and disease susceptibility. For example, we established consomic strains named “B6-ChrN^MSM^” (Takada et al. 2008) in which each chromosome of the B6 strain was replaced by its counterpart from MSM. Information on intersubspecific nucleotide variants between B6 and MSM can be used to associate genotypes to phenotypes or disease susceptibilities in these consomic strains. Currently, RIKEN BRC can provide both the mouse resource, B6-ChrN^MSM^, and the information on genomic variations that is required to exploit this mouse resource. Tools such as the Monarch Initiative (Mungall et al. 2017) and DisGeNET knowledge platform (Piñero et al. 2020) enable functional gene annotation from a database analysis and allow results from mouse genetic analyses to be translated into effects of human genomic variations on human phenotypes.

### Future perspectives

Because many mouse strains are used for modeling of human diseases, crosslinking to human databases is one of the important issues to be addressed for development of mouse genomic variation database. We plan to further improve MoG+ through increased collaboration with a human genomic variation database TogoVar (https://togovar.biosciencedbc.jp), which integrates multiple datasets from human variant databases such as GEM Japan Whole-Genome Aggregation (GEM-J WGA) Panel (https://www.amed.go.jp/en/aboutus/collaboration/ga4gh_gem_japan.html), JGA-NGS, JGA-SNP (https://humandbs.biosciencedbc.jp/en/), Exome Aggregation Consortium (ExAC: https://gnomad.broadinstitute.org), ClinVar (https://www.ncbi.nlm.nih.gov/clinvar/), Human Genetic Variation Database (HGVD: https://www.hgvd.genome.med.kyoto-u.ac.jp/download.html), and ToMMo 4.7KJPN Allele Frequency Panel (4.7KJPN: https://jmorp.megabank.tohoku.ac.jp/202001/downloads/#variant). Since basic gene functions are evolutionarily conserved and defects in orthologues may cause similar phenotypes in different organisms, MoG+ will be modified to integrate genetic variation data from other mammals and other vertebrates. The expansion of the database to include genome data in different taxa will be of value for exploring gene function using information of synteny in different organisms. Integration of data on genomic variation in different species will necessitate the development and use of common vocabularies such as Sequence Ontology (http://www.sequenceontology.org) and Variation Ontology (http://variationontology.org/instructions.shtml). Currently we are working on the collaboration to develop a common data format for genetic variation databases including MoG+ and TogoVar based on the resource description framework (RDF), a framework to provide a formal description of knowledge and common application programming interface (API) (World Wide Web Consortium 1999). Using common API, we plan to make it possible to simultaneously search mouse and human genomic variation data. Furthermore, we plan to integrate MoG+ with other RDF-based bioresource databases operated in the RIKEN BRC including bioresource, gene, orthology and ontology for phenotypes and diseases (Masuya et al 2021). Through further updates of data and improvements of software applications, we anticipate that MoG+ will become a powerful database for comparisons of orthologous genomic sequence between human and mouse, and for cross-species comparisons.

## Materials and methods

### Measurements of body size and physiological phenotypes of the Mishima Battery

An overview of the establishment of the Mishima Battery is presented in the Supplementary information (Figure S1) and has been described by Koide et al. (2000) and Furuse et al. (2002). The strains were established and maintained at the NIG (http://www.shigen.nig.ac.jp/mouse/strain/). At present, “Mishima Battery” mouse strains are distributed to the research community via RIKEN BRC (https://web.brc.riken.jp/en/) and NIG (https://shigen.nig.ac.jp/mouse/nig/). C57BL6/JJcl was purchased from CLEA Japan (Tokyo, Japan) and bred at the NIG. For measurement of all traits, mice 9–11 weeks of age (67–79 days after birth) were fasted from 6 to 10 am (± 30 min) prior to blood sampling. In total, 613 animals (300 males and 313 females) were used for body size and physiological measurements (Table S1). Environment and housing, and body size and physiological measurements were performed as described in our previous report (Takada et al. 2008). In brief, body weight (BW), total body length (TOTL; nose-to-tip of tail distance), body length (BL; nose-to-anus distance), and tail lengths (TL) were measured. Heart (HEA), liver (LIV), spleen (SPL), kidney (KID), and testis (TES) were dissected and weighed. Tissue weight was measured and represented as a percentage of BW excluding fat and internal organs. Dorsal and inguinal deposits of subcutaneous fat tissues (SFAT), perirenal, gonadal, and mesenteric deposits of visceral fat tissues (VFAT), and dorsal brown adipose tissues (DBAT) were dissected and weighed individually. The total weight of fat tissue was determined as a percentage of BW. Various blood characteristics were measured: inorganic phosphate (IP), HDL cholesterol, total cholesterol (TCH), and triglyceride (TG) were measured immediately after blood sampling using an automated dry chemistry analyzer (Drychem, Fuji-Film Medical); albumin (ALB), alkaline phosphatase (ALP), and amylase (AMY) were measured with a VetScan blood chemistry analyzer (Abaxis) using whole blood samples. Adiposity index (AI) = total fat tissue weight (g)/BW (g) × 100; body mass index (BMI) = BW/length2 × 100. The phenotypic data was analyzed using R (Ihaka and Gentleman 1996). Boxplots were constructed using the R graphics “boxplot()” command in the default setting. Statistical significance was determined in a *t*-test with Bonferroni correction using the R Stats Package among the Mishima Battery strains and B6. A measured value was considered significant if the corrected *t*-test *p* value was less than 0.05 and highly significant if the *p* value was less than 0.001. All animal experiments were approved by the Animal Care and Use Committee of NIG.

### Resequencing of the Mishima Battery

We resequenced genomic DNAs from the Mishima Battery mouse strains BLG2, HMI, NJL, PGN2, KJR, CHD, BFM, SWN, and MSM. Sequence data from JF1 (DRA000323) was obtained previously (Takada et al. 2013). All of these strains are maintained as pedigreed breeding stocks at the NIG. Samples used in the resequencing study are listed in Table S2. DNA sequencing was performed with an Hiseq2000 (Illumina) according to the manufacturer’s protocols. Raw reads were obtained from the whole-genome sequencing data and adaptor sequences and low-quality bases were removed using an in-house script before mapping. Low-quality sequences were defined by an averaged quality value (QV) (< 20 for a given base ± 1 adjacent nucleotide) and were marked. If a marked position was located at either the 5′ or 3′ end or both, the bases were trimmed. Finally, only high-quality paired-end reads with ≥ 20 nucleotides were selected. The mouse reference genome (mm10), which was soft-masked by RepeatMasker (http://www.repeatmasker.org), was obtained from the Project website (http://hgdownload.cse.ucsc.edu/goldenpath/mm10/chromosomes/) and used as the mapping reference. Filtered reads were aligned to the mouse reference genome by our in-house program, which was set at > 95% sequence identity (indels were treated as mismatches) with the reference sequence. Multi-mapping, where it is difficult to map a sequencing read to a particular position, is often the cause of incorrect genotyping. A read was considered as being multi-hit if the difference between the first and second highest sequence identities between the read and the reference genome was 2% or less. All other reads were regarded as uniquely mapped reads. Uniquely mapped pair read alignments were analyzed further. The alignments were converted from sequence alignment/map (SAM) format to sorted, indexed binary alignment/map (BAM) files (SAMtools; Li et al. 2009). The Picard tool (http://broadinstitute.github.io/picard/) was used to remove duplicate reads and the output BAM files were realigned using GATK IndelRealigner (https://gatk.broadinstitute.org/).

### Calling SNPs and short indels

The BAM files produced above were used for calling SNPs and short indels; SAMtools mpileup (Li et al. 2009) was used to output tsv, which was then converted to vcf for annotation. To detect authentic variants and to minimize false positives, target regions with high confidence of variant calling were defined by excluding genomic regions using various filter criteria. (1) Read depth. To filter out read depth outliers, the mean and standard deviation of read depth of each individual was calculated. Here, the minimum depth in the analysis conditions was set to 10 with an upper SD limit of 2.5. (2) Mapped-read balance. For allelic balance read mapping, at least 1 forward and 1 reverse read were used to map genomic regions. (3) Indels. Indels were annotated using GATK software tools (HaplotypeCaller) and the adjacent 10 bp were then excluded from target genomic regions (Note, this filter was not applied to indel calls). (4) Nucleotide types and strand bias. We retained variant sites that were covered by at least one read on the reference forward strand, reference reverse strand, alternative forward nucleotide types, and alternative reverse nucleotide types. For example, we retained SNPs A (20 forward reads, 12 reverse reads) and G (15 forward reads, 18 reverse reads) but discarded SNPs A (18 forward reads, 0 reverse reads) and G (19 forward reads, 22 reverse reads). All biased SNPs and their adjacent 10 bp sites were excluded from the genomic target regions. (5) Gaps. All variant sites located at the end of the read, with an average size from the end of read within 10 bp, were excluded from genomic target regions and their adjacent 10 bp sites were also excluded. A dendrogram was constructed from the entire Mishima Battery SNP set (strain name.snp.tsv; https://molossinus.brc.riken.jp/pub/Wild10_2020/) using the allele sharing distance (ASD) criterion. The dendrogram was explored using the general UPGMA methods. The analysis for acquisition of genomic data and the data analysis for the genomic variation detection and the dendrogram construction were performed at NIG and ROIS-DS.

### Note

In 2020, the BAM files produced above were used for calling SNPs and short indels using HaplotypeCaller implemented in GATK software tools (https://gatk.broadinstitute.org/). The filtering conditions for the nucleotide variant detection were the same as those described above. New vcf files are available in the download page (https://molossinus.brc.riken.jp/pub/Wild10_2020/).

### Comparison genomic variants of the Mishima Battery with public data

Nucleotide variant datasets of the Mishima Battery, consisting of pgn2v1, bfmv1, blg2v1, njlv1, chdv1, kjrv1, swnv1, hmiv1, msmv4, and jf1v3, respectively, were used to compare with dbSNPs (Mouse dbSNP 142; ftp://ftp-mouse.sanger.ac.uk/). The homozygous variants were used in this comparison (Low-quality regions around indels were also included). The count of variants was applied up to the third candidates. The results are downloadable from https://molossinus.brc.riken.jp/pub/Wild10_2020/.

## Supplementary Information

Below is the link to the electronic supplementary material.

Figure S1. The Mishima Battery of wild-derived inbred strains. The figure illustrates strains belonging to *M. m. domesticus*, *M. m. musculus*, *M. m. castaneus*, and *M. m. molossinus* in that order from top to bottom. For each image, brightness, orientation, and size may have been altered. No other modifications have been made. PGN2 is an inbred mouse strain of the subspecies *M. m. domesticus*. PGN2 is descended from wild mice caught by Dr. P. Michael in the farm of a Mr. J. Pigeon located about 21 km south of Windsor, Ontario, Canada in 1978. In 1979, founder mice (3 female and 2 male) were donated to Dr. Kazuo Moriwaki of NIG by Dr. P. Michael. BFM is an inbred mouse strain of the subspecies *M. m. domesticus*. This strain was established from a few wild mice trapped by Dr. F. Bonhomme on the University Campus of Montpellier, France in 1976. In 1980, founder mice (3 female and 3 male) were donated to Dr. Kazuo Moriwaki of NIG by Dr. F. Bonhomme. BLG2 is an inbred mouse strain of the subspecies *M. m. musculus*. This strain was established from wild mice trapped near General Toshevo in Bulgaria in 1980 by Dr. F. Bonhomme; the mice were brother-sister mated at the Institut Pasteur. In 1981, founder mice (2 female and 2 male) were donated to Dr. Kazuo Moriwaki of NIG by Dr. F. Bonhomme. NJL is an inbred mouse strain of the subspecies *M. m. musculus*. This strain was established from wild mice trapped by Dr. J.P. Hjorth 15km west of Aarhus in 1980. In 1980, founder mice (6 female and 4 male) were donated to Dr. Kazuo Moriwaki of NIG from by Dr. J.P. Hjorth. CHD is an inbred mouse strain of the subspecies *M. m. musculus*. This strain was established from wild mice trapped in Chengdu, China. In 1981, founder mice (3 female and 3 male) were transferred to Dr. Kazuo Moriwaki of NIG.KJR is an inbred mouse strain of the subspecies *M. m. musculus*. This strain was established from wild mice trapped on Kojuri Island, Republic of Korea. In 1984, founder mice (2 female and 1 male) were transferred to Dr. Kazuo Moriwaki of NIG. SWN is an inbred mouse strain of the subspecies *M. m. musculus*. This strain was established from wild mice trapped on the campus of Agricultural College, Suweon, Republic of Korea. In 1984, founder mice (3 female and 1 male) were transferred to Dr. Kazuo Moriwaki of NIG by Dr. Cho. HMI is an inbred mouse strain of the subspecies *M. m. castaneus*. HMI is descended from wild mice trapped in He-mei, Taiwan by Dr. Sakaizumi in 1986. Founder mice (4 female and 3 male) were given to Dr. Kazuo Moriwaki of NIG by Dr. Sakaizumi in the same year. MSM is an inbred mouse strain of the subspecies *M. m. molossinus*, collected in 1978 in Mishima, Shizuoka-ken, Japan (Moriwaki et al. 1994, 2009). The divergence time between *M. m. molossinus* and the west European subspecies *M. m. domesticus*, from which most classical laboratory strains are derived, is estimated to be roughly one million years (Yonekawa et al. 1980; Moriwaki 1994, 2009). JF1 is an inbred mouse strain of the subspecies *M. m. molossinus*. The ancestors of JF1 were found at a pet market in Denmark and transferred to NIG in 1987, where they were established as an inbred strain in 1993. Figure S2. Box-plots of body size, tissue weights, and physiological characteristics of Mishima Battery strains. (a) BW: Body weight, (b) TOTL: Total body length, (c) BL: Body length, (d) TL: Tail length, (e) BMI: Body mass index, (f) TES: Testis, (g) SPL: Spleen, (h) LIV: Liver, (i) KID: Kidneys, (j) HEA: Heart, (k) VFAT: Visceral fat tissues, (l) SFAT: Subcutaneous fat tissues, (m) DBAT: Dorsal brown adipose tissues, (n) AI: Adiposity index. Blood concentrations of selected physiological characteristics: (o) IP: Inorganic phosphate, (p) HDL: High-density lipoprotein cholesterol, (q) TCH: Total cholesterol, (r) TG: Triglyceride, (s) ALB: Albumin, (t) ALP: Alkaline phosphatase, and (u) AMY: Amylase. Figure S3. Trait-strain matrices of the Mishima Battery strains. Significance levels between strains in the Mishima Battery were confirmed using a Bonferroni correction to account for multiple-hypothesis testing. A measured value was considered significant if the corrected *t*-test P value was less than 0.05 (pale red) and highly significant if the P-value was less than 0.001 (red). (a) BW: Body weight, (b) TOTL: Total body length, (c) BL: Body length, (d) TL: Tail lengths, (e) BMI: Body mass index, (f) TES: Testis, (g) SPL: Spleen, (h) LIV: Liver, (i) KID: Kidneys, (j) HEA: Heart, (k) VFAT: Visceral fat tissues, (l) SFAT: Subcutaneous fat tissues, (m) DBAT: Dorsal brown adipose tissues, (n) AI: Adiposity index, (o) IP: Inorganic phosphate, (p) HDL: High-density lipoprotein cholesterol, (q) TCH: Total cholesterol, (r) TG: Triglyceride, (s) ALB: Albumin, (t) ALP: Alkaline phosphatase, and (u) AMY: Amylase. Figure S4. Dendrogram of the Mishima Battery constructed using the whole set of SNP variants. Low-quality regions around indels were excluded in this analysis. Supplementary file1 (PDF 6397 KB)

## Data Availability

The sequence data from this study have been submitted to the DDBJ Sequence Read Archive (DRA) (http://trace.ddbj.nig.ac.jp/dra/index_e.shtml) under accession numbers DRA001112 for BLG2, DRA001111 for HMI, DRA001129 for NJL, DRA001130 for PGN2, DRA001123 for KJR, DRA001124 for CHD, DRA001125 for BFM, DRA001126 for SWN, and DRA002726 for MSM. The nucleotide variant data from this study have been deposited in the European Variation Archive (EVA) (https://www.ebi.ac.uk/eva) under accession number PRJEB48602. The BAM files containing the sequences of all the strains and the lists of nucleotide variants and all Supplemental Materials used in this study are available on the download page of MoG+ (https://molossinus.brc.riken.jp/pub/Wild10_2020/).
