# Supplementary material for: MoG+: a database of genomic variations across three mouse subspecies for biomedical research
Source: Mamm Genome. 2021 Nov 15;33(1):31–43. doi: 10.1007/s00335-021-09933-w (PMC8913468; doi:10.1007/s00335-021-09933-w)

Table S1. Samples used in the phenotyping study

Strains	Subspecies	Male	DAB*	Female	DAB*
PGN2/Ms	*Domesticus*	19	68-76	16	70-76
BFM/Ms	*Domesticus*	18	70-76	15	70-76
BLG2/Ms	*Musculus*	15	72-76	15	72-76
NJL/Ms	*Musculus*	16	70-74	17	69-75
CHD/Ms	*Musculus*	15	70-77	16	70-77
SWN/Ms	*Musculus*	15	68-79	18	70-79
KJR/Ms	*Musculus*	15	70-76	16	70-76
MSM/Ms	*Molossinus*	72	70-76	78	70-76
JF1/Ms	*Molossinus*	77	70-76	65	70-76
HMI/Ms	*Castaneus*	16	70-76	18	67-76
C57BL/6JJcl	Experimental strain	38	69-73	37	69-73
Total		300	68-79	313	69-79

* Days after birth at the time used for the analysis

Table S2. Identities of the mice used in the sequencing study

STRAIN	ID	SEX	DOB*	DOSC†	F‡
PGN2/Ms	422a	Female	2010.11.1	2011.7.14	F74
BFM/2Ms	388f	Female	2010.8.10	2011.7.19	F106
BLG2/Ms	518a	Female	2010.12.25	2011.2.21	F89
NJL/Ms	357a	Female	2011.5.13	2011.7.20	F76
CHD/Ms	473d	Female	2011.5.9	2011.7.20	F75
KJR/Ms	791c	Female	2011.5.12	2011.7.21	F79
SWN/Ms	465a	Female	2010.9.6	2011.7.13	F61
HMI/Ms	477a	Female	2010.11.4	2011.2.24	F61
MSM/Ms	1509a	Female	2008.11.24	2009.2.5	F85
JF1/Ms§	75a	Female	2009.09.21	2009.12.3	F28

* Date of birth

† Date of sample collection

‡ The number of inbreeding generations the time used for the analysis

§ Published data (Takada et al. 2013)

Table S3. Summary of the resequencing data showing the filtered genomic variations in each strain

Strain	PGN2/Ms	BFM/Ms	BLG2/Ms	NJL/Ms	CHD/Ms	KJR/Ms	SWN/Ms	HMI/Ms	MSM/Ms	JF1/Ms
Subspecies	*Domesticus*	*Domesticus*	*Musculus*	*Musculus*	*Musculus*	*Musculus*	*Musculus*	*Castaneus*	*Molossinus*	*Molossinus*
Summary of the resequencing										
Read depth (mean)	40.5	38.4	32.5	31.6	33.6	28.8	38.0	26.5	78.9	46.1
Read depth (sd)	14.2	14.3	12.9	12.6	15.0	12.4	16.7	10.4	32.0	18.1
Effective genome length (A)	2,096,595,900	2,059,694,138	1,897,688,219	1,965,472,791	1,944,662,689	1,929,922,677	1,954,314,656	1,888,303,969	1,991,769,970	1,923,645,511
Effective genome length (X)	127,730,084	125,667,682	114,377,959	118,099,252	117,578,154	117,086,080	118,383,751	115,517,619	117,086,080	115,617,797
Effective genome length (Total)	2,224,325,984	2,185,361,820	2,012,066,178	2,083,572,043	2,062,240,843	2,047,008,757	2,072,698,407	2,003,821,588	2,108,856,050	2,039,263,308
Percent genome cover (w/a gap)	86.9	85.4	78.6	81.4	80.6	80.0	81.0	78.3	82.4	79.7
Percent genome cover (w/a gap & repeat)	95.0	93.9	89.0	91.4	90.6	90.3	90.9	89.0	92.1	90.6
Single nucleotide polymorphisms*										
SNPs (A)	5,669,047	6,135,913	13,258,522	13,735,885	14,218,479	13,514,652	14,477,603	13,456,125	16,277,466	14,334,422
hSNPs (A)	193,357	168,567	161,414	207,906	194,643	171,800	211,140	175,571	295,939	230,013
Total SNPs (A)	5,862,404	6,304,480	13,419,936	13,943,791	14,413,122	13,686,452	14,688,743	13,631,696	16,573,405	14,564,435
Percent SNPs (A)	0.28	0.31	0.71	0.71	0.74	0.71	0.75	0.72	0.83	0.76
Percent hSNPs (A)	0.01	0.01	0.01	0.01	0.01	0.01	0.01	0.01	0.01	0.01
SNPs (X)	122,773	140,473	616,974	644,204	641,128	624,859	651,648	578,675	697,381	648,322
hSNPs (X)	14,756	13,220	14,831	19,037	23,256	20,471	25,644	15,256	34,377	24,217
SNPs (A+X)	5,791,820	6,276,386	13,875,496	14,380,089	14,859,607	14,139,511	15,129,251	14,034,800	16,974,847	14,982,744
hSNPs (A+X)	208,113	181,787	176,245	226,943	217,899	192,271	236,784	190,827	330,316	254,230
Total SNPs (X)	137,529	153,693	631,805	663,241	664,384	645,330	677,292	593,931	731,758	672,539
Total SNPs (A+X))	5,999,933	6,458,173	14,051,741	14,607,032	15,077,506	14,331,782	15,366,035	14,225,627	17,305,163	15,236,974
Percent SNPs (X)	0.11	0.12	0.55	0.56	0.57	0.55	0.57	0.51	0.61	0.58
Percent hSNPs (X)	0.01	0.01	0.01	0.02	0.02	0.02	0.02	0.01	0.03	0.02
Percent SNPs (Total)	0.27	0.30	0.70	0.70	0.73	0.70	0.74	0.71	0.82	0.75
Patterns of nucleotide substitution†										
Ti (A)	3,966,939	4,272,451	9,110,247	9,458,556	9,770,829	9,286,696	9,956,971	9,253,492	11,206,004	9,874,378
Tv (A)	1,895,465	2,032,029	4,309,689	4,485,235	4,642,293	4,399,756	4,731,772	4,378,204	5,367,401	4,690,057
Ti+Tv (A)	5,862,404	6,304,480	13,419,936	13,943,791	14,413,122	13,686,452	14,688,743	13,631,696	16,573,405	14,564,435
Ti /Tv (A)	2.09	2.10	2.11	2.11	2.10	2.11	2.10	2.11	2.09	2.11
Ti (X)	85,387	95,966	403,435	423,243	422,965	410,964	430,438	379,170	463,111	427,476
Tv (X)	52,142	57,727	228,370	239,998	241,419	234,366	246,854	214,761	268,647	245,063
Ti+Tv (X)	137,529	153,693	631,805	663,241	664,384	645,330	677,292	593,931	731,758	672,539
Ti /Tv (X)	1.64	1.66	1.77	1.76	1.75	1.75	1.74	1.77	1.72	1.74
INDELs										
Insertion (A)	412,014	445,161	799,465	817,497	837,719	812,705	833,323	828,746	956,161	762,911
Hetero Insertion (A)	160,144	164,374	240,280	226,433	223,033	217,589	236,111	241,199	318,241	212,730
Total Insertion (A)	572,158	609,535	1,039,745	1,043,930	1,060,752	1,030,294	1,069,434	1,069,945	1,274,402	975,641
Deletion (A)	528,046	573,662	1,130,505	1,150,744	1,190,853	1,159,629	1,197,920	1,086,382	1,342,645	1,043,251
Hetero Deletion (A)	157,652	159,793	227,402	212,804	210,269	203,846	218,055	208,529	290,838	198,488
Total Deletion (A)	685,698	733,455	1,357,907	1,363,548	1,401,122	1,363,475	1,415,975	1,294,911	1,633,483	1,241,739
Insertion (X)	16,131	17,837	43,546	44,889	44,813	44,109	44,897	43,484	49,192	41,647
Hetero Insertion (X)	7,834	8,422	13,013	12,587	12,712	12,754	13,591	12,917	17,292	12,849
Total Insertion (X)	23,965	26,259	56,559	57,476	57,525	56,863	58,488	56,401	66,484	54,496
Deletion (X)	20,277	22,685	63,589	65,312	65,400	64,637	66,114	55,730	70,804	59,934
Hetero Deletion (X)	8,658	9,034	14,149	13,831	14,410	14,457	15,100	12,213	19,013	13,535
Total Deletion (X)	28,935	31,719	77,738	79,143	79,810	79,094	81,214	67,943	89,817	73,469
Total Insertion (A+X)	596,123	635,794	1,096,304	1,101,406	1,118,277	1,087,157	1,127,922	1,126,346	1,340,886	1,030,137
Total Deletion (A+X)	714,633	765,174	1,435,645	1,442,691	1,480,932	1,442,569	1,497,189	1,362,854	1,723,300	1,315,208

* hSNP; Heterozygous SNP
A; Autosomal chromosome
X; X chromosome
† Ti; Transition
Tv; Transversion

Table S4. Allele-sharing distances among the Mishima battery strains

	C57BL/6J (mm10)	PGN2/Ms	BFM/Ms	BLG2/Ms	NJL/Ms	CHD/Ms	KJR/Ms	SWN/Ms	HMI/Ms	MSM/Ms
PGN2/Ms	0.1655									
BFM/Ms	0.1873	0.1689								
BLG2/Ms	0.4339	0.4703	0.4719							
NJL/Ms	0.4397	0.4727	0.4749	0.1835						
CHD/Ms	0.4593	0.4974	0.4994	0.1931	0.1912					
KJR/Ms	0.4544	0.4954	0.4974	0.1867	0.1847	0.1117				
SWN/Ms	0.4554	0.4954	0.4985	0.1879	0.1864	0.1121	0.0686			
HMI/Ms	0.4564	0.4784	0.4824	0.4504	0.4542	0.4407	0.4452	0.4447		
MSM/Ms	0.4494	0.4937	0.4958	0.1928	0.1973	0.1257	0.0913	0.0922	0.4457	
JF1/Ms	0.4428	0.4879	0.491	0.2053	0.2079	0.1406	0.1118	0.1136	0.4295	0.1018

Table S5. Summary of the annotated genomic variations in the database*

Strain		PGN2/Ms		BFM/Ms		BLG2/Ms		NJL/Ms		CHD/Ms		KJR/Ms		SWN/Ms		HMI/Ms		MSM/Ms		JF1/Ms	
Variant varsion in the database		pgn2v1		bfmv1		blg2v1		njlv1		chdv1		kjrv1		swnv1		hmiv1		msmv4		jf1v3	
		Variants rate details																			
Chromoson	Length	Variants	Percent	Variants	Percent	Variants	Percent	Variants	Percent	Variants	Percent	Variants	Percent	Variants	Percent	Variants	Percent	Variants	Percent	Variants	Percent
1	195,471,971	787,093	0.4	913,289	0.47	1,803,005	0.92	1,819,111	0.93	1,942,906	0.99	1,915,299	0.98	1,913,805	0.98	1,899,378	0.97	1,507,842	0.77	1,873,373	0.96
2	182,113,224	637,124	0.35	719,579	0.4	1,613,193	0.89	1,643,087	0.9	1,702,159	0.93	1,687,577	0.93	1,684,469	0.92	1,702,205	0.93	1,461,054	0.8	1,648,557	0.91
3	160,039,680	624,331	0.39	680,139	0.42	1,537,776	0.96	1,549,410	0.97	1,632,353	1.02	1,616,125	1.01	1,616,306	1.01	1,616,371	1.01	1,362,815	0.85	1,583,650	0.99
4	156,508,116	598,924	0.38	690,112	0.44	1,436,800	0.92	1,447,640	0.92	1,495,282	0.96	1,488,100	0.95	1,472,363	0.94	1,482,211	0.95	1,223,678	0.78	1,454,769	0.93
5	151,834,684	530,558	0.35	610,349	0.4	1,482,124	0.98	1,512,811	1	1,562,339	1.03	1,551,656	1.02	1,549,925	1.02	1,517,518	1	1,330,535	0.88	1,528,546	1.01
6	149,736,546	616,652	0.41	706,153	0.47	1,377,880	0.92	1,415,182	0.95	1,474,070	0.98	1,454,407	0.97	1,471,045	0.98	1,504,180	1	1,228,137	0.82	1,422,450	0.95
7	145,441,459	527,832	0.36	563,834	0.39	1,263,873	0.87	1,308,582	0.9	1,377,194	0.95	1,355,478	0.93	1,366,558	0.94	1,316,574	0.91	1,144,050	0.79	1,332,457	0.92
8	129,401,213	637,124	0.49	677,179	0.52	1,090,000	0.84	1,069,497	0.83	1,122,047	0.87	1,098,501	0.85	1,096,766	0.85	1,235,482	0.95	917,956	0.71	824,658	0.64
9	124,595,110	464,145	0.37	519,021	0.42	1,161,017	0.93	1,153,260	0.93	1,209,821	0.97	1,202,417	0.97	1,198,520	0.96	1,206,397	0.97	1,034,926	0.83	1,178,897	0.95
10	130,694,993	462,217	0.35	534,857	0.41	1,313,756	1.01	1,347,554	1.03	1,388,386	1.06	1,373,456	1.05	1,370,139	1.05	1,345,728	1.03	1,080,183	0.83	1,346,330	1.03
11	122,082,543	453,844	0.37	502,601	0.41	1,144,349	0.94	1,152,205	0.94	1,191,275	0.98	1,183,035	0.97	1,180,347	0.97	1,178,592	0.97	942,039	0.77	1,149,682	0.94
12	120,129,022	502,406	0.42	571,393	0.48	1,065,083	0.89	1,056,028	0.88	1,095,181	0.91	1,057,357	0.88	1,068,801	0.89	1,105,487	0.92	837,137	0.7	1,042,305	0.87
13	120,421,639	442,340	0.37	517,000	0.43	1,106,913	0.92	1,134,952	0.94	1,181,337	0.98	1,160,943	0.96	1,158,954	0.96	1,173,320	0.97	988,612	0.82	1,129,657	0.94
14	124,902,244	546,484	0.44	600,228	0.48	1,027,536	0.82	1,045,502	0.84	1,065,210	0.85	1,043,662	0.84	1,046,766	0.84	1,107,020	0.89	891,487	0.71	992,694	0.79
15	104,043,685	339,653	0.33	392,844	0.38	1,026,521	0.99	1,036,575	1	1,094,081	1.05	1,090,463	1.05	1,092,398	1.05	1,058,880	1.02	935,762	0.9	1,076,343	1.03
16	98,207,768	329,486	0.34	389,821	0.4	953,310	0.97	956,894	0.97	1,009,526	1.03	1,000,692	1.02	1,006,511	1.02	990,917	1.01	855,718	0.87	986,232	1
17	94,987,271	383,607	0.4	395,453	0.42	892,861	0.94	864,915	0.91	962,547	1.01	949,503	1	948,887	1	931,186	0.98	789,691	0.83	909,294	0.96
18	90,702,639	303,567	0.33	350,820	0.39	904,034	1	927,686	1.02	958,032	1.06	951,336	1.05	952,379	1.05	923,220	1.02	826,471	0.91	941,589	1.04
19	61,431,566	225,203	0.37	251,281	0.41	594,314	0.97	587,367	0.96	638,880	1.04	634,473	1.03	635,829	1.04	613,993	1	541,616	0.88	626,238	1.02
X	171,031,299	238,061	0.14	277,927	0.16	1,012,621	0.59	1,047,212	0.61	1,060,208	0.62	1,050,871	0.61	1,059,042	0.62	965,446	0.56	899,731	0.53	1,024,657	0.6
Total	2,633,776,672	9,650,651	0.37	10,863,880	0.41	23,806,966	0.9	24,075,470	0.91	25,162,834	0.96	24,865,351	0.94	24,889,810	0.95	24,874,105	0.94	20,799,440	0.79	24,072,378	0.91

Number variantss by type

Type	Total	Total	Total	Total	Total	Total	Total	Total	Total	Total
SNP	7,696,121	8682908	19,913,083	20259896	21175757	20911580	20976631	20962576	17727646	20,369,496
MNP	0	0	0	0	0	0	0	0	0	0
INS†	932,680	1032702	1,816,613	1793279	1852240	1837627	1824789	1896491	1348488	1,724,702
DEL†	1,021,850	1148270	2,077,270	2022295	2134837	2116144	2088390	2015038	1723306	1,978,180
MIXED	0	0	0	0	0	0	0	0	0	0
INTERVAL	0	0	0	0	0	0	0	0	0	0
Total	9,650,651	10863880	23,806,966	24075470	25162834	24865351	24889810	24874105	20799440	24,072,378

Number of effects by impact

Type (alphabetical order)	Count	Percent	Count	Percent	Count	Percent	Count	Percent	Count	Percent	Count	Percent	Count	Percent	Count	Percent	Count	Percent	Count	Percent
HIGH	2041	0	2349	0	4242	0	4153	0	4385	0	4288	0	4406	0	4567	0	3684	0	4,175	0
LOW	89001	0	101798	0	227752	0	230609	0	239523	0	237811	0	238038	0	240922	0	224507	0	231,741	0
MODERATE	37247	0	42816	0	79669	0	82282	0	86665	0	85152	0	85809	0	83738	0	76210	0	81,776	0
MODIFIER	20961633	0.99	23703595	0.99	52663275	0.99	53347447	0.99	55715240	0.99	55137787	0.99	55123002	0.99	55176887	0.99	47041138	0.99	53,376,198	0.99

Number of effects by functional class

Type (alphabetical order)	Count	Percent	Count	Percent	Count	Percent	Count	Percent	Count	Percent	Count	Percent	Count	Percent	Count	Percent	Count	Percent	Count	Percent
MISSENSE	36229	0.35	41526	0.35	77481	0.31	80017	0.32	84236	0.32	82662	0.32	83438	0.32	81591	0.31	74479	0.3	79,599	0.31
NONSENSE	319	0	332	0	459	0	584	0	577	0	551	0	588	0	498	0	423	0	544	0
SILENT	66173	0.64	75502	0.64	170585	0.69	172919	0.68	180019	0.68	178454	0.68	178633	0.68	180786	0.69	169734	0.69	174,210	0.68

Number of effects by type and region

Type (alphabetical order)	Count	Percent	Count	Percent	Count	Percent	Count	Percent	Count	Percent	Count	Percent	Count	Percent	Count	Percent	Count	Percent	Count	Percent
3_prime_UTR_variant	123,853	0.01	137,547	0.01	310,662	0.01	311,169	0.01	326,315	0.01	322,340	0.01	322,744	0.01	323,839	0.01	301,113	0.01	313,426	0.01
5_prime_UTR_premature_start_codon_gain_variant	2,331	0	2,962	0	6,375	0	6,597	0	6,829	0	6,975	0	6,899	0	6,701	0	6,618	0	6,574	0
5_prime_UTR_truncation	1	0	1	0	5	0	2	0	2	0	4	0	3	0	-	-	2	0	1	0
5_prime_UTR_variant	21,481	0	26,582	0	53,133	0	54,884	0	56,955	0	57,891	0	57,914	0	56,302	0	54,657	0	54,108	0
disruptive_inframe_deletion	424	0	473	0	917	0	910	0	1,003	0	1,079	0	1,026	0	948	0	765	0	975	0
disruptive_inframe_insertion	288	0	414	0	626	0	667	0	730	0	719	0	699	0	613	0	453	0	599	0
downstream_gene_variant	1,900,026	0.09	2,129,936	0.09	4,591,790	0.09	4,653,212	0.09	4,863,165	0.09	4,809,265	0.09	4,813,181	0.09	4,871,589	0.09	4,111,175	0.09	4,659,315	0.09
exon_loss_variant	1	0	1	0	5	0	2	0	2	0	4	0	3	0	-	-	2	0	1	0
frameshift_variant	707	0	821	0	1,497	0	1,322	0	1,399	0	1,391	0	1,461	0	1,554	0	1,198	0	1,394	0
inframe_deletion	212	0	265	0	428	0	487	0	492	0	446	0	440	0	431	0	420	0	429	0
inframe_insertion	230	0	297	0	510	0	498	0	544	0	559	0	532	0	531	0	370	0	476	0
initiator_codon_variant	16	0	11	0	14	0	16	0	15	0	11	0	12	0	16	0	12	0	16	0
intergenic_region	5,655,949	0.27	6,365,985	0.27	13,710,510	0.26	13,885,949	0.26	14,516,018	0.26	14,318,970	0.26	14,351,223	0.26	14,301,022	0.26	11,686,469	0.25	13,862,905	0.26
intragenic_variant	1,879	0	1,774	0	5,195	0	5,480	0	5,384	0	5,355	0	5,483	0	5,161	0	4,336	0	5,265	0
intron_variant	11,217,008	0.53	12,737,993	0.53	29,113,639	0.55	29,490,839	0.55	30,786,282	0.55	30,520,205	0.55	30,462,355	0.55	30,426,030	0.55	26,513,397	0.56	29,551,000	0.55
missense_variant	36,102	0	41,381	0	77,216	0	79,754	0	83,935	0	82,388	0	83,138	0	81,255	0	74,226	0	79,324	0
non_coding_exon_variant	156,814	0.01	178,885	0.01	403,709	0.01	407,229	0.01	425,276	0.01	421,538	0.01	422,675	0.01	426,227	0.01	380,757	0.01	410,860	0.01
non_coding_transcript_variant	74	0	95	0	170	0	151	0	177	0	184	0	184	0	156	0	130	0	154	0
splice_acceptor_variant	515	0	563	0	1,041	0	1,072	0	1,160	0	1,107	0	1,143	0	1,144	0	936	0	1,045	0
splice_donor_variant	406	0	510	0	998	0	917	0	947	0	968	0	926	0	1,044	0	891	0	934	0
splice_region_variant	22,877	0	25,976	0	56,501	0	56,857	0	58,846	0	58,428	0	58,651	0	59,346	0	53,654	0	56,849	0
start_lost	70	0	74	0	150	0	156	0	170	0	167	0	185	0	183	0	146	0	177	0
stop_gained	334	0	352	0	494	0	608	0	604	0	580	0	610	0	520	0	453	0	563	0
stop_lost	73	0	105	0	186	0	174	0	212	0	189	0	191	0	230	0	176	0	176	0
stop_retained_variant	49	0	46	0	129	0	130	0	141	0	155	0	149	0	127	0	121	0	134	0
synonymous_variant	66,123	0	75,455	0	170,456	0	172,788	0	179,878	0	178,299	0	178,484	0	180,659	0	169,613	0	174,076	0
upstream_gene_variant	1,905,056	0.09	2,148,160	0.09	4,525,197	0.09	4,589,454	0.09	4,788,218	0.09	4,734,264	0.09	4,739,579	0.09	4,820,019	0.09	4,037,030	0.09	4,569,949	0.09

Type (alphabetical order)	Count	Percent	Count	Percent	Count	Percent	Count	Percent	Count	Percent	Count	Percent	Count	Percent	Count	Percent	Count	Percent	Count	Percent
DOWNSTREAM	1,900,026	0.09	2,129,934	0.09	4,591,786	0.09	4,653,209	0.09	4,863,162	0.09	4,809,265	0.09	4,813,179	0.09	4,871,581	0.09	4,111,174	0.09	4,659,312	0.09
EXON	258,169	0.01	294,948	0.01	648,369	0.01	656,726	0.01	685,966	0.01	679,208	0.01	681,305	0.01	685,065	0.01	621,189	0.01	661,156	0.01
INTERGENIC	5,655,949	0.27	6,365,985	0.27	13,710,510	0.26	13,885,949	0.26	14,516,018	0.26	14,318,970	0.26	14,351,223	0.26	14,301,022	0.26	11,686,469	0.25	13,862,905	0.26
INTRON	11,198,192	0.53	12,716,553	0.53	29,066,859	0.55	29,443,916	0.55	30,737,888	0.55	30,472,084	0.55	30,414,102	0.55	30,376,721	0.55	26,469,110	0.56	29,504,183	0.55
SPLICE_SITE_ACCEPTOR	510	0	557	0	1,028	0	1,066	0	1,155	0	1,100	0	1,137	0	1,140	0	934	0	1,039	0
SPLICE_SITE_DONOR	388	0	494	0	975	0	898	0	927	0	942	0	903	0	1,020	0	864	0	911	0
SPLICE_SITE_REGION	22,019	0	24,977	0	54,690	0	55,000	0	56,833	0	56,468	0	56,613	0	57,397	0	51,923	0	54,926	0
TRANSCRIPT	1,953	0	1,869	0	5,365	0	5,631	0	5,561	0	5,539	0	5,667	0	5,317	0	4,466	0	5,419	0
UPSTREAM	1,905,056	0.09	2,148,160	0.09	4,525,197	0.09	4,589,454	0.09	4,788,218	0.09	4,734,264	0.09	4,739,579	0.09	4,820,019	0.09	4,037,030	0.09	4,569,949	0.09
UTR_3_PRIME	123,850	0.01	137,546	0.01	310,659	0.01	311,168	0.01	326,315	0.01	322,340	0.01	322,742	0.01	323,834	0.01	301,110	0.01	313,422	0.01
UTR_5_PRIME	23,810	0	29,535	0	59,500	0	61,474	0	63,770	0	64,858	0	64,805	0	62,998	0	61,270	0	60,668	0

* Low-quality regions around indels were included in this analysis

† Max 46bp

Table S6. Comparison of the data in MoG+ with publicly available genome variation data*

	SNP †	SNP(%)	Insertion †	Insertion (%)	Deletion †	Deletion (%)
Number of coordinates used in the analysis	81,866,820		6,100,247		8,046,754	
Polymorphic only in the Mishima Battery strains	8,062,070	9.8	1,308,960	21.5	1,610,909	20.0
Genomic variations only in‡						
Domesticus	11,681	0.0001	960	0.0002	1,716	0.0002
Musculus	27,459	0.0335	2,538	0.0416	4,318	0.0537
Castaneus	2,226,674	2.7199	131,181	2.1504	210,955	2.6216
Molossinus	38,127	0.0466	1,651	0.0271	3,476	0.0432
Intra-subspecific genomic variations between						
BFM/Ms-PGN2/Ms	7,265,319	8.9	675,129	11.1	794,142	9.9
BFM/Ms-WSB/EiJ	7,841,482	9.6	709,175	11.6	911,704	11.3
PGN2/Ms-WSB/EiJ	7,121,105	8.7	662,778	10.9	836,683	10.4
CHD/Ms-BLG2/Ms	8,264,995	10.1	803,409	13.2	1,041,941	12.9
CHD/Ms-KJR/Ms	4,649,177	5.7	569,685	9.3	715,027	8.9
CHD/Ms-NJL/Ms	8,164,587	10.0	796,263	13.1	1,032,268	12.8
CHD/Ms-SWN/Ms	4,664,651	5.7	562,086	9.2	709,939	8.8
CHD/Ms -PWK/Ms	9,711,682	11.9	964,423	15.8	1,230,077	15.3
BLG2/Ms -KJR/Ms	8,002,616	9.8	786,301	12.9	1,019,499	12.7
BLG2/Ms -NJL/Ms	7,856,475	9.6	752,546	12.3	956,672	11.9
BLG2/Ms -SWN/Ms	8,041,478	9.8	783,461	12.8	1,017,471	12.6
BLG2/Ms -PWK/PhJ	9,407,873	11.5	921,131	15.1	1,139,920	14.2
KJR/Ms -NJL/Ms	7,890,133	9.6	777,886	12.8	1,006,707	12.5
KJR/Ms -SWN/Ms	2,774,652	3.4	401,881	6.6	477,516	5.9
KJR/Ms -PWK/Ms	9,480,279	11.6	946,277	15.5	1,204,567	15.0
NJL/Ms -SWN/Ms	7,953,169	9.7	773,317	12.7	1,002,074	12.5
NJL/Ms -PWK/PhJ	9,169,275	11.2	913,867	15.0	1,122,727	14.0
SWN/Ms -PWK/PhJ	9,496,185	11.6	945,024	15.5	1,201,931	14.9
HMI/Ms-CAST/EiJ	14,427,289	17.6	1,218,981	20.0	1,484,053	18.4
JF1/Ms-MSM/Ms	5,891,707	7.2	1,098,252	18.0	1,377,925	17.1
JF1/Ms-MOLF/EiJ	9,461,164	11.6	863,421	14.2	1,046,391	13.0
MSM/Ms-MOLF/EiJ	10,487,467	12.8	1,304,350	21.4	1,637,277	20.3

* Mouse dbSNP 142

† Genotypic differences in the coordinates

% Percent of SNP variants between the number of coordinates (81,866,820) and each the strains or subspecies group

$ For the Mishima battery's data, low-quality regions around indels were included in this analysis, and homozygous genotypes were used for variants count.

‡ Subspecies are grouped as follows:

Domesticus ; BFM/Ms, PGN2/Ms and WSB/EiJ

Musculus ; BLG2/Ms, NJL/Ms, CHD/Ms, KJR/Ms, SWN/Ms and PWK/PhJ

Castaneus ; HMI/Ms and CAST/EiJ

Molossinus ; JF1/Ms, MSM/Ms and MOLF/EiJ

Takada_Figure S1

Mus musculus domesticus

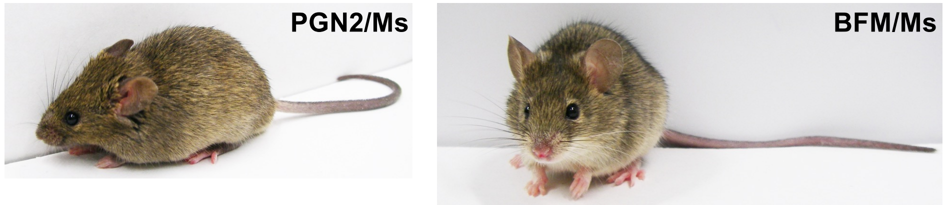

Mus musculus musculus

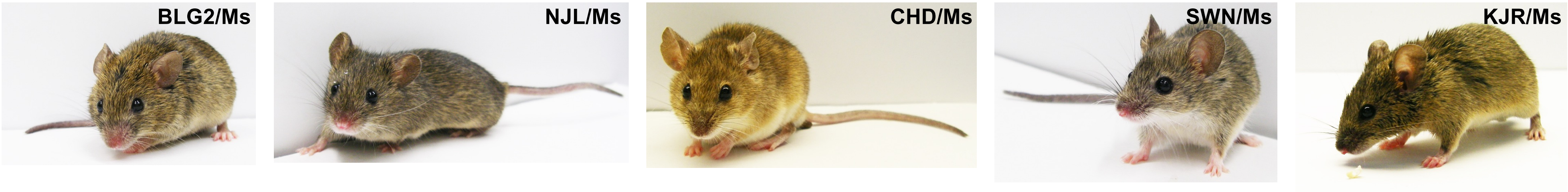

Mus musculus castaneus

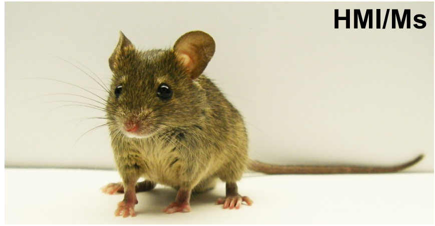

Mus musculus molossinus

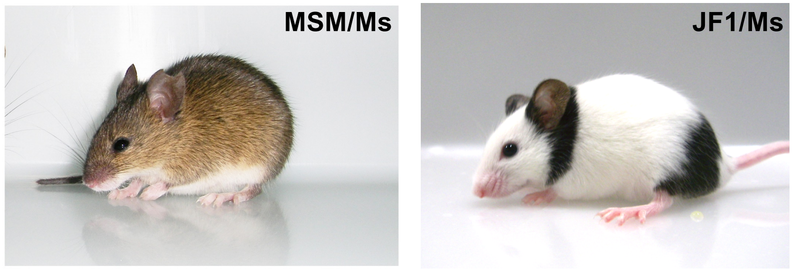

Takada_Figure S2

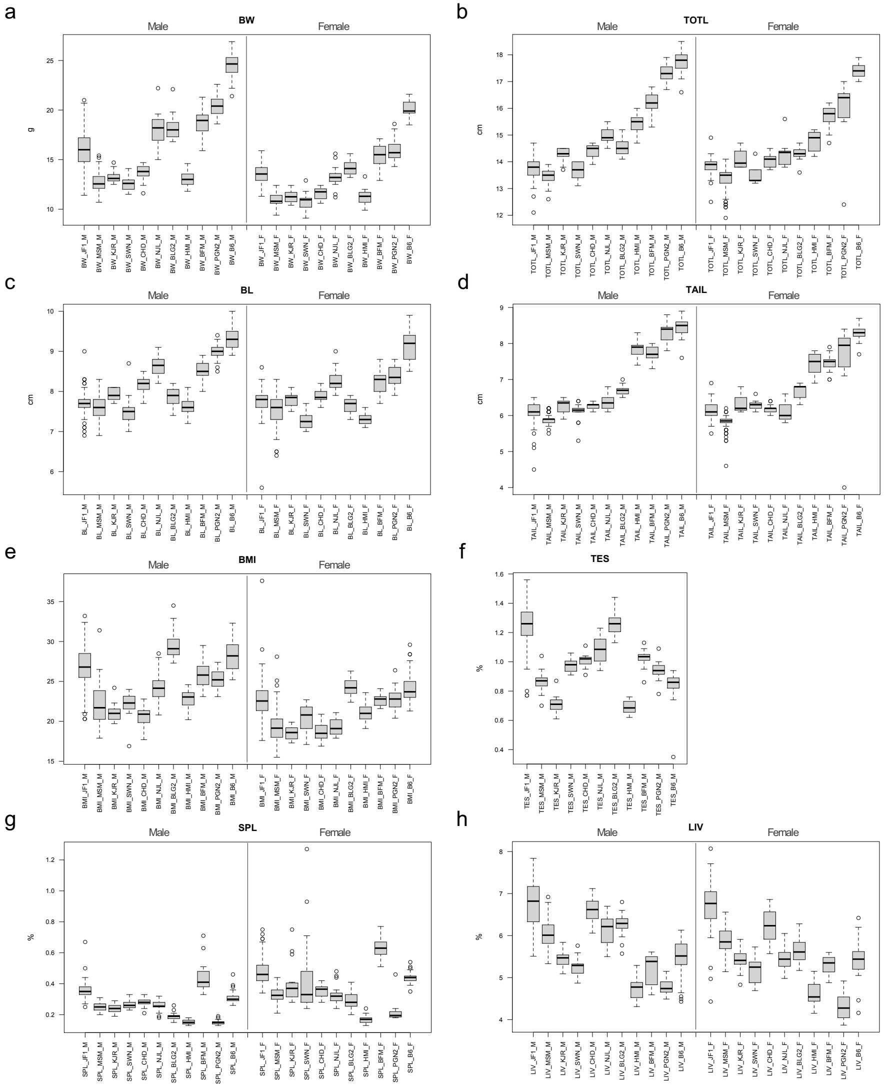

Takada_Figure S2_continued

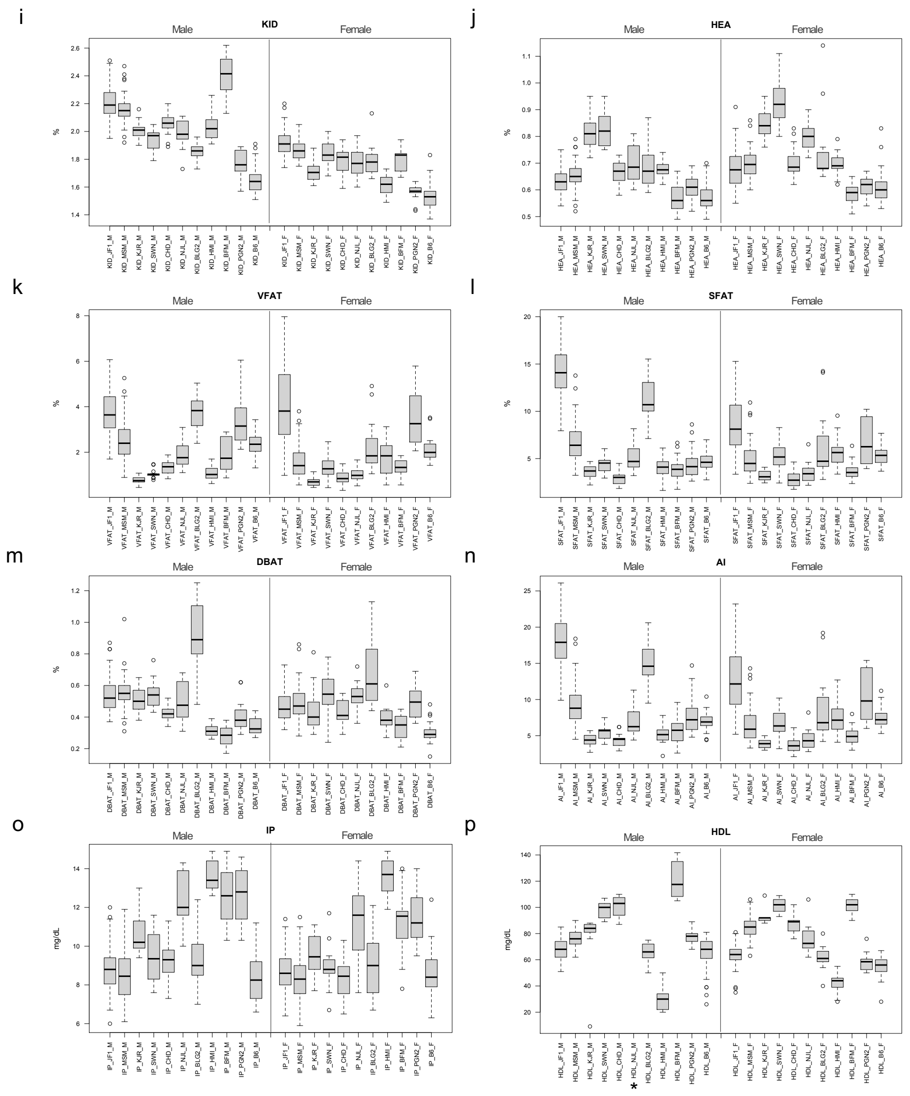

Takada_Figure S2_continued

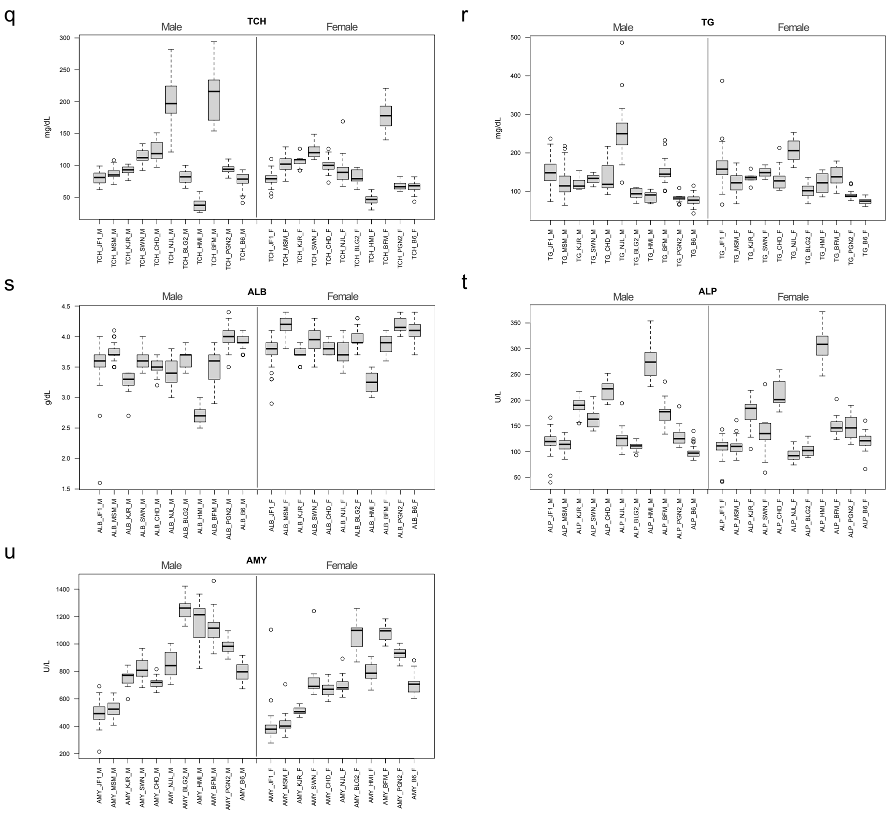

Takada_Figure S3

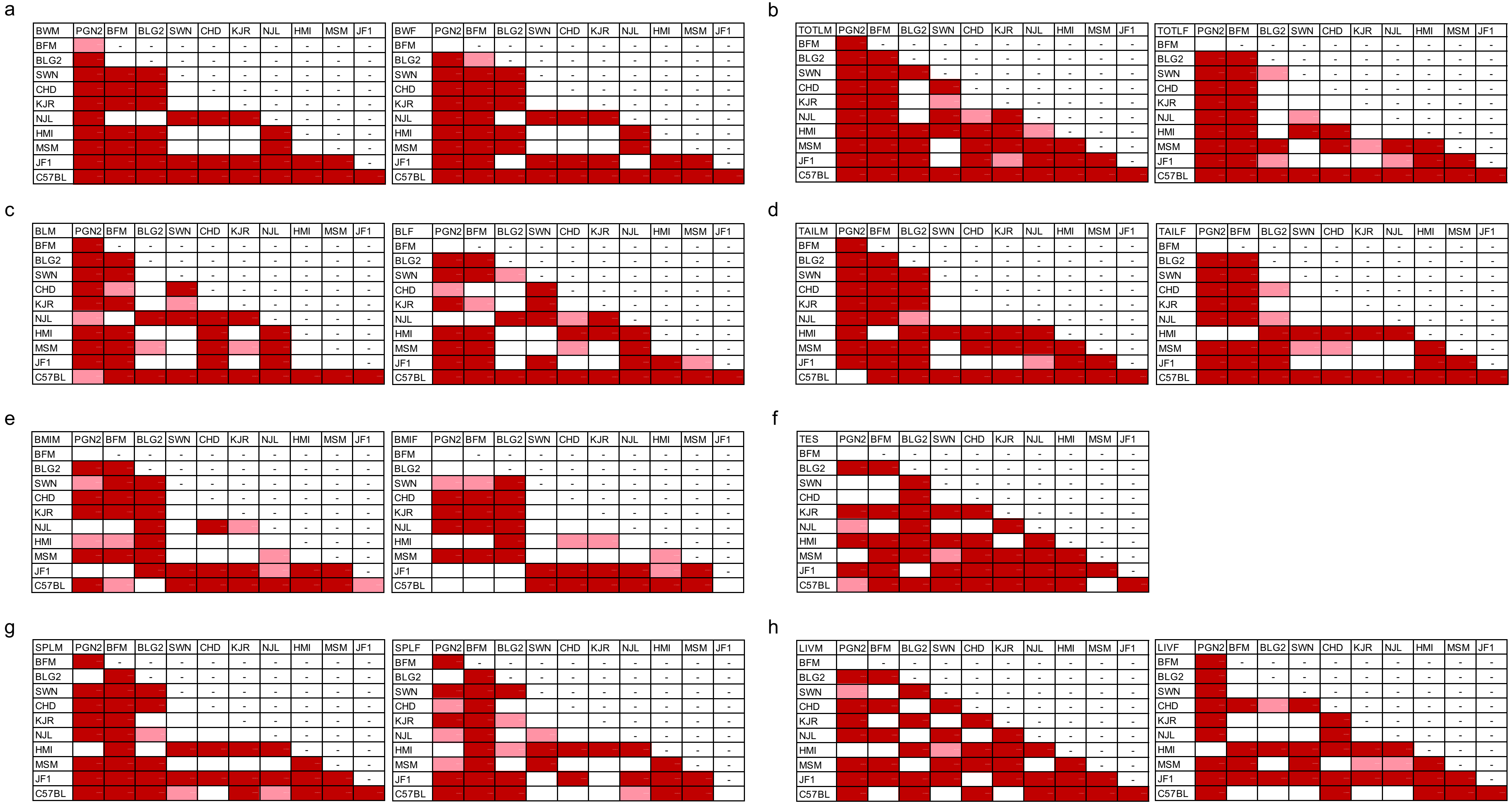

Takada_Figure S3_continued

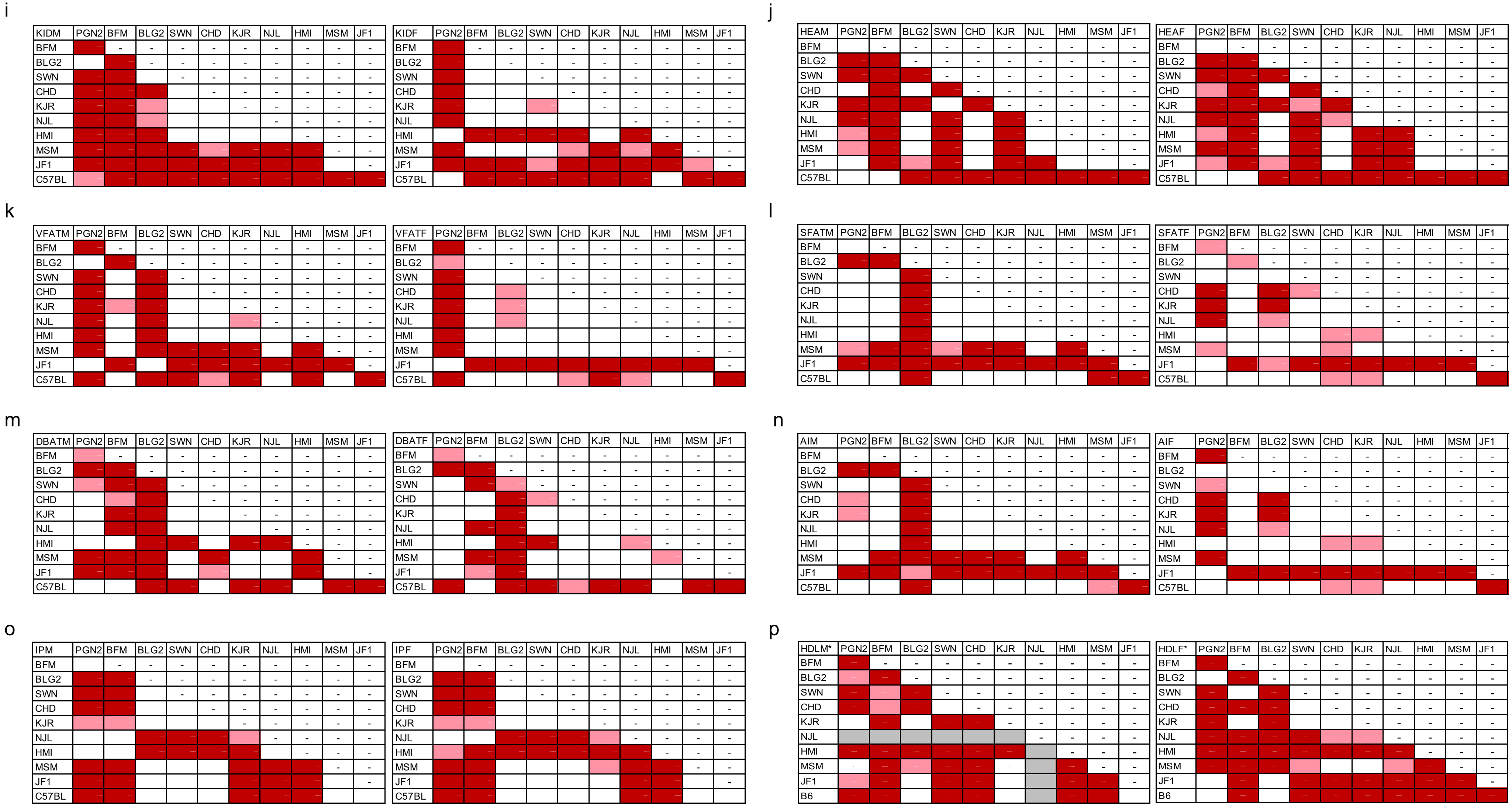

Takada_Figure S3_continued

q

TCHM	PGN2	BFM	BLG2	SWN	CHD	KJR	NJL	HMI	MSM	JF1
BFM		-	-	-	-	-	-	-	-	-
BLG2			-	-	-	-	-	-	-	-
SWN				-	-	-	-	-	-	-
CHD					-	-	-	-	-	-
KJR						-	-	-	-	-
NJL							-	-	-	-
HMI								-	-	-
MSM									-	-
JF1										-
C57BL										

TCHF	PGN2	BFM	BLG2	SWN	CHD	KJR	NJL	HMI	MSM	JF1
BFM		-	-	-	-	-	-	-	-	-
BLG2			-	-	-	-	-	-	-	-
SWN				-	-	-	-	-	-	-
CHD					-	-	-	-	-	-
KJR						-	-	-	-	-
NJL							-	-	-	-
HMI								-	-	-
MSM									-	-
JF1										-
C57BL										

r

TGM	PGN2	BFM	BLG2	SWN	CHD	KJR	NJL	HMI	MSM	JF1
BFM		-	-	-	-	-	-	-	-	-
BLG2			-	-	-	-	-	-	-	-
SWN				-	-	-	-	-	-	-
CHD					-	-	-	-	-	-
KJR						-	-	-	-	-
NJL							-	-	-	-
HMI								-	-	-
MSM									-	-
JF1										-
C57BL										

TGF	PGN2	BFM	BLG2	SWN	CHD	KJR	NJL	HMI	MSM	JF1
BFM		-	-	-	-	-	-	-	-	-
BLG2			-	-	-	-	-	-	-	-
SWN				-	-	-	-	-	-	-
CHD					-	-	-	-	-	-
KJR						-	-	-	-	-
NJL							-	-	-	-
HMI								-	-	-
MSM									-	-
JF1										-
C57BL										

s

ALBM	PGN2	BFM	BLG2	SWN	CHD	KJR	NJL	HMI	MSM	JF1
BFM		-	-	-	-	-	-	-	-	-
BLG2			-	-	-	-	-	-	-	-
SWN				-	-	-	-	-	-	-
CHD					-	-	-	-	-	-
KJR						-	-	-	-	-
NJL							-	-	-	-
HMI								-	-	-
MSM									-	-
JF1										-
C57BL										

ALBF	PGN2	BFM	BLG2	SWN	CHD	KJR	NJL	HMI	MSM	JF1
BFM		-	-	-	-	-	-	-	-	-
BLG2			-	-	-	-	-	-	-	-
SWN				-	-	-	-	-	-	-
CHD					-	-	-	-	-	-
KJR						-	-	-	-	-
NJL							-	-	-	-
HMI								-	-	-
MSM									-	-
JF1										-
C57BL										

t

ALPM	PGN2	BFM	BLG2	SWN	CHD	KJR	NJL	HMI	MSM	JF1
BFM		-	-	-	-	-	-	-	-	-
BLG2			-	-	-	-	-	-	-	-
SWN				-	-	-	-	-	-	-
CHD					-	-	-	-	-	-
KJR						-	-	-	-	-
NJL							-	-	-	-
HMI								-	-	-
MSM									-	-
JF1										-
C57BL										

ALPF	PGN2	BFM	BLG2	SWN	CHD	KJR	NJL	HMI	MSM	JF1
BFM		-	-	-	-	-	-	-	-	-
BLG2			-	-	-	-	-	-	-	-
SWN				-	-	-	-	-	-	-
CHD					-	-	-	-	-	-
KJR						-	-	-	-	-
NJL							-	-	-	-
HMI								-	-	-
MSM									-	-
JF1										-
C57BL										

u

AMYM	PGN2	BFM	BLG2	SWN	CHD	KJR	NJL	HMI	MSM	JF1
BFM		-	-	-	-	-	-	-	-	-
BLG2			-	-	-	-	-	-	-	-
SWN				-	-	-	-	-	-	-
CHD					-	-	-	-	-	-
KJR						-	-	-	-	-
NJL							-	-	-	-
HMI								-	-	-
MSM									-	-
JF1										-
C57BL										

AMYF	PGN2	BFM	BLG2	SWN	CHD	KJR	NJL	HMI	MSM	JF1
BFM		-	-	-	-	-	-	-	-	-
BLG2			-	-	-	-	-	-	-	-
SWN				-	-	-	-	-	-	-
CHD					-	-	-	-	-	-
KJR						-	-	-	-	-
NJL							-	-	-	-
HMI								-	-	-
MSM									-	-
JF1										-
C57BL										

Takada_Figure S4

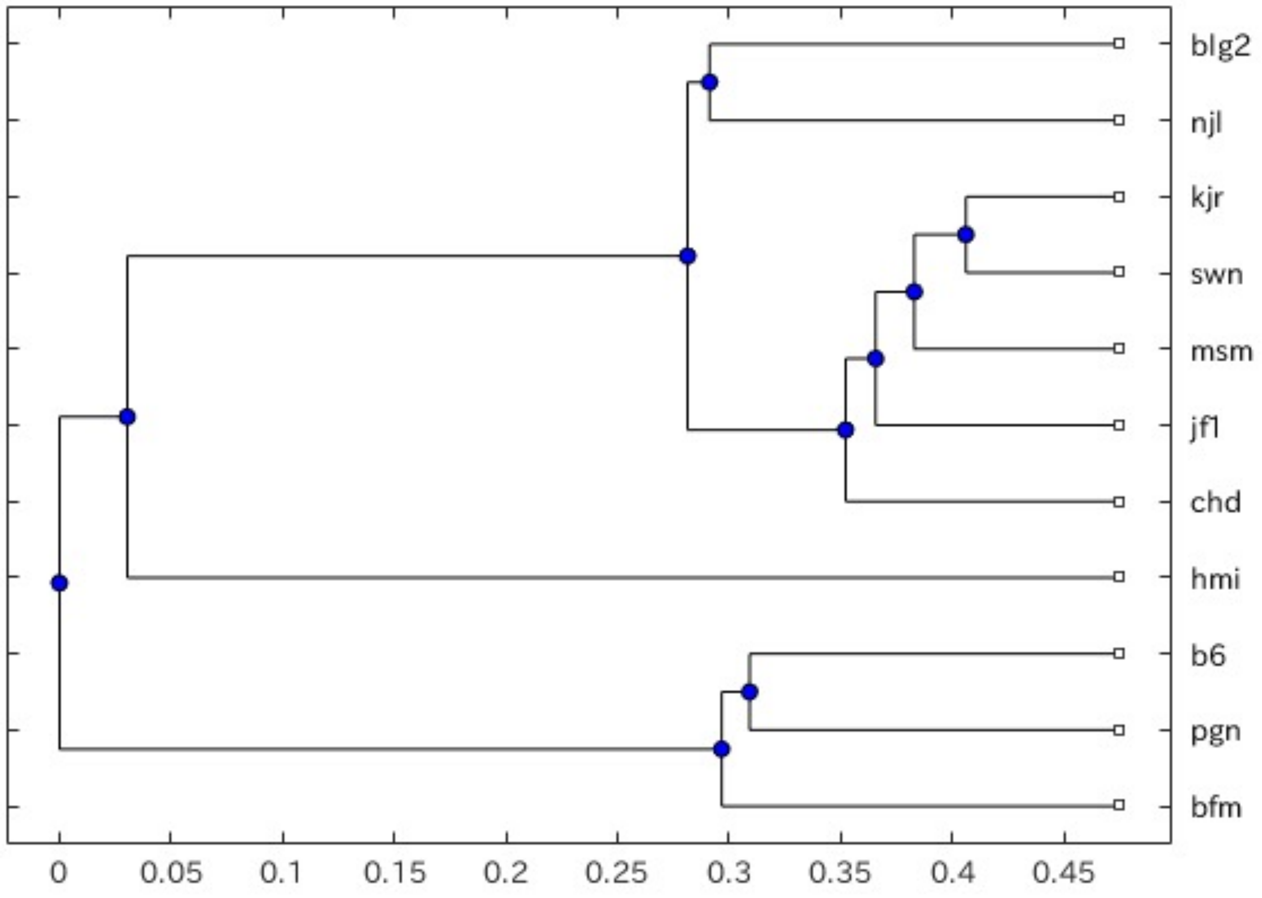

Supplement: Supplementary file 1 — Figure S1. The Mishima Battery of wild-derived inbred strains. The figure illustrates strains belonging to M. m. domesticus, M. m. musculus, M. m. castaneus, and M. m. molossinus in that order from top to bottom. For each image, brightness, orientation, and size may have been altered. No other modifications have been made. PGN2 is an inbred mouse strain of the subspecies M. m. domesticus. PGN2 is descended from wild mice caught by Dr. P. Michael in the farm of a Mr. J. Pigeon located about 21 km south of Windsor, Ontario, Canada in 1978. In 1979, founder mice (3 female and 2 male) were donated to Dr. Kazuo Moriwaki of NIG by Dr. P. Michael. BFM is an inbred mouse strain of the subspecies M. m. domesticus. This strain was established from a few wild mice trapped by Dr. F. Bonhomme on the University Campus of Montpellier, France in 1976. In 1980, founder mice (3 female and 3 male) were donated to Dr. Kazuo Moriwaki of NIG by Dr. F. Bonhomme. BLG2 is an inbred mouse strain of the subspecies M. m. musculus. This strain was established from wild mice trapped near General Toshevo in Bulgaria in 1980 by Dr. F. Bonhomme; the mice were brother-sister mated at the Institut Pasteur. In 1981, founder mice (2 female and 2 male) were donated to Dr. Kazuo Moriwaki of NIG by Dr. F. Bonhomme. NJL is an inbred mouse strain of the subspecies M. m. musculus. This strain was established from wild mice trapped by Dr. J.P. Hjorth 15km west of Aarhus in 1980. In 1980, founder mice (6 female and 4 male) were donated to Dr. Kazuo Moriwaki of NIG from by Dr. J.P. Hjorth. CHD is an inbred mouse strain of the subspecies M. m. musculus. This strain was established from wild mice trapped in Chengdu, China. In 1981, founder mice (3 female and 3 male) were transferred to Dr. Kazuo Moriwaki of NIG.KJR is an inbred mouse strain of the subspecies M. m. musculus. This strain was established from wild mice trapped on Kojuri Island, Republic of Korea. In 1984, founder mice (2 female and 1 male) were tran [file 335_2021_9933_MOESM1_ESM.pdf]
